# Concordance between target trial emulation and randomised controlled trials: systematic review and meta-analysis

**DOI:** 10.1136/bmj-2025-086810

**Published:** 2026-05-19

**Authors:** Canlong Wang, Dongfeng Tang, Peter von Dadelszen, Chengsheng Ju, Lu Liu, Yanzhong Wang, Laura A Magee

**Affiliations:** 1 School of Life Course and Population Sciences, King's College London, London, UK; 2 Department of Obstetrics and Gynaecology, University of British Columbia, Vancouver, BC, Canada; 3 Research Department of Practice and Policy, School of Pharmacy, University College London, London, UK

## Abstract

**Objectives:**

To assess the concordance of results between paired target trial emulations and their benchmarking randomised controlled trials and to explore determinants of emulation success.

**Design:**

Systematic review and meta-analysis.

**Data sources:**

Medline, EMBASE, Scopus, PsycINFO, and Web of Science (2010 to 2025).

**Study selection:**

Observational studies that explicitly aimed to emulate target randomised controlled trials of a health intervention were included.

**Data extraction:**

Data were extracted on study domain, target population, intervention, comparator, and main outcomes for each emulation and corresponding randomised controlled trial.

**Main outcome measures:**

For 21 predefined emulation design features, concordance was assessed using Pearson correlation coefficients, standardised difference agreement, and ratio of ratios, with subgroup and regression analyses of study characteristics and concordance.

**Results:**

Among 106 target trial emulation-randomised controlled trial pairs, the Pearson correlation coefficient was 0.58 (95% confidence interval (CI) 0.43 to 0.69), standardised difference agreement was 79% (84/106), and summary ratio of ratios was 0.97 (95% CI 0.92 to 1.03; I^2^=42%). In 62 pairs with closer emulation of trial design, a higher level of agreement was observed, with a Pearson correlation coefficient of 0.83 (0.73 to 0.89) and standardised difference agreement of 87% (54/62). In subgroup analysis, trial emulations tended to systematically underestimate treatment effects from randomised controlled trials for specific outcomes related to venous thromboembolism and major adverse cardiovascular events, while overestimating those for respiratory outcomes, with pooled ratio of ratios of 0.76 (0.58 to 1.00; I^2^=40%), 0.91 (0.85 to 0.98; I^2^=35%), and 1.20 (1.03 to 1.40; I^2^=40%), respectively. Moreover, emulations based on claims data tended to underestimate the treatment effects (ratio of ratios 0.89, 0.81 to 0.98; I^2^=39%). In exploratory univariate regression analyses, poorer target trial emulation-randomised controlled trial concordance was associated with emulation designs with imbalanced baseline population characteristics, delayed treatment effect, and lower outcome emulation quality.

**Conclusions:**

Current target trial emulations achieve moderate concordance when replicating their corresponding randomised controlled trials. Concordance could be improved by better emulation design, including better baseline and outcome emulation and leveraging multisource linked databases.

**Systematic review registration:**

PROSPERO CRD42024619811.

## Introduction

The randomised controlled trial is considered to be the strongest form of evidence for evaluating the effectiveness of health interventions. However, randomised controlled trials are sometimes not feasible, owing to ethical considerations, logistical challenges, or resource constraints.^1^ As such, causal inferences are often drawn using observational data.^2^

Target trial emulation helps investigators to clearly specify the causal estimand and avoid design related biases (for example, immortal time bias and other selection biases).^3^ ^4^ Using target trial emulation, observational studies have been shown to yield treatment effect estimates similar to those of the corresponding randomised controlled trials.^5^ A pragmatic workflow involves benchmarking an emulation against a high quality randomised controlled trial to assess concordance, then extending the same protocolised design to underrepresented populations (for example, older adults, multimorbidity, pregnancy), with a pre-specified estimand and transparent reporting of deviations from the randomised controlled trial protocol.^6^ The increasing popularity of target trial emulation as an approach to drawing causal inference from observational data raises questions about the utility and scope across different settings.

Although many emulation studies have been published across various fields, how closely they replicate effect estimates from randomised controlled trials and the study characteristics associated with concordance remain unclear. In a 2023 systematic review of 200 emulation studies, narrative synthesis described inconsistent reporting, with only 58% of emulations fully describing their protocol details.^7^ In a quantitative analysis of three healthcare claims databases used to emulate 32 highly selected randomised controlled trials, Wang and colleagues found strong correlation (Pearson’s r=0.82) among these target trial emulation-randomised controlled trial pairs^5^; in a post hoc, exploratory analysis of eight factors, concordance in the pairs was higher in those classified as having closer emulation of the randomised controlled trial design. In a subsequent analysis of 29 of these 32 target trial emulation-randomised controlled trial pairs, Heyard and colleagues identified that three of Wang and colleagues’ eight potential explanatory factors explained a substantial proportion of the differences between the concordance results: treatment started in hospital (which does not appear in claims data), discontinuation of some baseline treatments at randomisation (which would be unusual in routine clinical care), and delayed onset of drug effects (which would be underreported in observational data).^8^ Although notable, these findings were based on the same three claims databases and a highly selected set of 32 trials, focusing exclusively on drug interventions. As a result, whether the observed concordance patterns generalise across diverse data sources, intervention types, and clinical domains remains unclear; the overall validity and generalisability remain uncertain and warrant a systematic review with quantitative assessment.

This review aims to comprehensively and quantitatively assess the degree of concordance between target trial emulations and their corresponding target randomised controlled trials and to explore study features that may systematically influence success and should be reported alongside study findings.

## Methods

This review is reported according to the Preferred Reporting Items for Systematic Reviews and Meta-analyses (PRISMA) reporting guidelines and was prospectively registered in the International Prospective Register of Systematic Reviews (PROSPERO, CRD42024619811).

### Searches

We searched Medline, EMBASE, Scopus, PsycINFO, and Web of Science for potentially eligible observational studies published between 1 January 2010 and 1 October 2025, using search terms such as “emulat* trial” and “target trial”. The search was limited to articles published in English. For details of the search strategy, see supplementary appendix 1.

### Eligibility criteria

We included studies if they were published in English and explicitly aimed (as stated in the abstract and/or methods) to emulate a benchmarking target trial of a health intervention, defined as the use of drugs, surgical procedures, or medical devices. We placed no restrictions on the therapeutic area of the study, geographical region, or data source. We excluded studies if they involved emulations of hypothetical trials or emulations that did not specify the randomised controlled trial being emulated; interventions that were behavioural, lifestyle, policy, financial, or educational, because these interventions are often complex, context dependent, and difficult to standardise or measure accurately in real world settings; and studies that did not report a ratio outcome as either a primary or a secondary outcome metric.

### Screening and data extraction

Two reviewers (CW and DT) independently screened the identified records for eligibility, at the level of title and abstract, followed by full text review. Disagreements were resolved through discussion, if needed. We did not mask reviewers to the journal article or study authors.

To explore individual studies’ risk of bias, and concordance between target trial emulation-randomised controlled trial pairs, the following information was extracted, independently, with any differences resolved by consensus: characteristics of the subject field, study population, intervention, comparator, and outcomes (table 1). When multiple emulation estimates were reported, the benchmarking estimate most closely aligned to the index randomised controlled trial eligibility criteria and follow-up window was extracted. Firstly, we assessed six criteria defining “close emulation” in the framework proposed by Wang and colleagues and applied by Heyard and colleagues^5^ ^8^ (that is, comparator emulation quality, outcome emulation quality, mixing of the effects of randomisation and discontinuation of baseline maintenance therapy, start of follow-up in hospital, run-in that selects responders to only one treatment arm in the randomised controlled trial, and delayed effect of the intervention); for detailed definitions, see supplementary appendix 2. In addition, we included two baseline population characteristics of relevance to population comparability: the absolute difference in mean age and the difference in sex distribution between the target trial emulation and the randomised controlled trial. Finally, we predefined and extracted 10 exploratory, study level design features (table 1).

**Table 1:** Predefined characteristics of target trial emulation protocols used to assess concordance with randomised controlled trials

Category	Variable
Participant characteristics (n=2)	Difference in age; difference in percentage of sex
Intervention characteristics (n=6)	Discontinuation of maintenance*; run-in to one arm*; treatment started in hospital*; delayed treatment effect*; dose titration; type of intervention
Control treatment characteristics (n=2)	Comparator emulation* †; placebo control
Outcome characteristics (n=1)	Outcome emulation* ‡
Exploratory design characteristics (n=10)	Nature of primary outcome; type of database used for emulation; database region; confounding adjustment methods; estimand (ATT or ATE); disease domain; corresponding trial design (non-inferiority or superiority); reporting metrics; whether protocol registered; whether report use of guideline

ATE=average treatment effect; ATT=average treatment effect on treated.

* These factors were used to assess “close emulation” emulation quality, with criteria adapted from Wang et al.^5^ Operationally, close emulation was defined as comparator and outcome emulation rated at least moderate, with at least one classified as good and none of: discontinuation of maintenance, run-in to one arm, treatment started in hospital, and delayed effect.

† Comparator emulation quality was classified as: “good” if randomised controlled trial (RCT) used active comparator and emulation applied same or clinically similar active drug; “moderate” if RCT used placebo and emulation used proxy or inactive comparator; and “poor” if non-equivalent comparator was used.

‡ Outcome emulation quality was classified as: “good” if outcome was reliably identifiable in claims/electronic health record data with high specificity and completeness; “moderate” if outcome had lower specificity or high missingness; and “poor” if outcome could not be directly captured or only weak proxies were available.

### Risk of bias assessment

For randomised controlled trials, two reviewers assessed risk of bias by using the Cochrane Risk of Bias Tool version 2.0 (RoB 2),^9^ according to five domains: randomisation process, deviations from intended interventions, outcome measurement, missing outcome data, and selection of the reported result. A study was judged to be at high risk of bias if at least one domain was assessed as being at high risk or if three or more domains were assessed as having some concerns. A study was judged to be at low risk of bias only if all domains were rated as low risk. In all other cases, the study was classified as having some concerns regarding the overall risk of bias.

For target trial emulations, we assessed risk of bias by using the Cochrane Risk Of Bias In Non-randomised Studies of Interventions (ROBINS-I) tool,^10^ according to seven domains: selection of participants, confounding, classification of interventions, deviations from intended interventions, missing data, outcome measurement, and selection of the reported result. A study was rated as low risk if all domains were judged to be at low risk, moderate if all domains were at low or moderate risk, serious if at least one domain was judged to be at serious risk, and critical if at least one domain was at critical risk of bias.

### Outcomes

To ensure comparability across studies with varying disease topics, we extracted ratio-type effect measures (for example, hazard ratio, risk ratio, or odds ratio) for each study’s primary outcome or the data needed to calculate it. When the ratio measure was not used to report the primary outcome, we selected key results from secondary outcomes. For example, in the PreVent emulation study,^11^ the primary outcome was the lowest oxygen saturation (continuous); we selected the hazard ratio corresponding to the secondary outcome of oxygen saturation <80%.

#### Agreement metrics between target trial emulation and randomised controlled trial

Of primary interest was agreement between effect estimates from each target trial emulation-randomised controlled trial pair, using eight published metrics. The four binary metrics were: standardised difference agreement, defined by standardised differences <1.96, as described by Wang and colleagues^5^; estimate agreement, defined by whether the point estimate from the target trial emulation fell within the 95% confidence interval for the randomised controlled trial results; full agreement for statistical significance, defined by estimates and confidence intervals on the same side of the null; and partial agreement for statistical significance, defined as meeting pre-specified non-inferiority criteria, even though the database study may have indicated superiority.

The four continuous metrics were: Pearson coefficient; intraclass correlation coefficient; relative per cent deviation of the ratio point estimate (defined as the per cent difference of the target trial emulation estimate relative to the matched randomised controlled trial); and ratio of ratios,^12^ ^13^ ^14^ ^15^ using the randomised controlled trial as the reference category, with ratio of ratios >1.0 indicating that the target trial emulation found higher odds of the outcome evaluated than the randomised controlled trial, and ratio of ratios <1.0 indicating that the target trial emulation found lower odds.

### Data analysis

We cleaned and analysed data in R version 4.2.0. All statistical analyses required that the effect estimates from target trial emulations and randomised controlled trials were approximately normally distributed, so we applied log transformations to the ratio results, as necessary. We summarised categorical variables with counts and percentages and continuous variables were summarised with mean and standard deviation or median and interquartile range, as appropriate for normally or non-normally distributed data.

We defined acceptable target trial emulation-randomised controlled trial concordance as: a standardised difference |Z| <1.96, reflecting differences within expected random variation^16^; Pearson coefficients interpreted as values <0.5 indicating poor, 0.5-0.75 moderate, 0.75-0.9 good, and >0.90 excellent agreement^17^; any deviation of the target trial emulation point estimate that is within 20% of the corresponding randomised controlled trial estimate, reflecting clinically acceptable equivalence^18^; and a ratio of ratios lower than the arbitrary threshold of 0.70 (or larger than the reciprocal, 1.43) indicating large differences in the magnitude of effect estimates.^19^ Additionally, we used Bland-Altman plots as a visual measure of consistency.

To ensure consistency in meta-analysis, we converted effect estimates for favourable outcomes (for example, survival) to unfavourable ones (for example, mortality).^12^ ^13^ The threshold for statistical significance was P<0.05. To account for potential heterogeneity and clustering of studies originating from the same study, we used a multilevel modelling approach, using the “metafor” package in R^20^; this allows for incorporation of random effects at multiple levels, facilitating the adjustment of both within study and between study variance components. To reduce the complexity of regression and avoid overfitting, we did leave-one-out cross validation.^21^

We did several sensitivity analyses to validate the robustness of the findings. These included: excluding influential outliers; excluding target trial emulation using randomised trial datasets; excluding trials at high risk of bias; excluding all target trial emulation-randomised controlled trial pairs from the RCT-DUPLICATE^5^; excluding target trial emulation-randomised controlled trial pairs with a >20% difference in sex proportions, as our exploratory analysis suggested that such imbalance may lead to poorer concordance; excluding studies reporting risk ratios; and, to overcome potential non-independence from multiple pairs per study, repeating the above six analyses in study independent subsets by retaining one pair per study—specifically, the pair with the minimum standardised difference (and, in a sensitivity analysis, the maximum standardised difference) within each study.

To investigate the association between emulation design features and concordance of target trial emulation-randomised controlled trial pairs, we used three complementary approaches: subgroup meta-analyses to assess variation in ratio of ratios across different design strata; linear mixed effects models using the log ratio between target trial emulation and randomised controlled trial estimates as the dependent variable, with random intercepts to account for study level clustering and multiple comparison adjustments (Benjamini-Hochberg false discovery rate and Bonferroni corrections); and standard difference as a descriptive measure of variability in concordance across strata.

### Patient and public involvement

No patients or members of the public were involved in the design, conduct, reporting, or dissemination plans of this research, as the study was based on secondary analysis of published studies.

## Results

**Fig 1:**
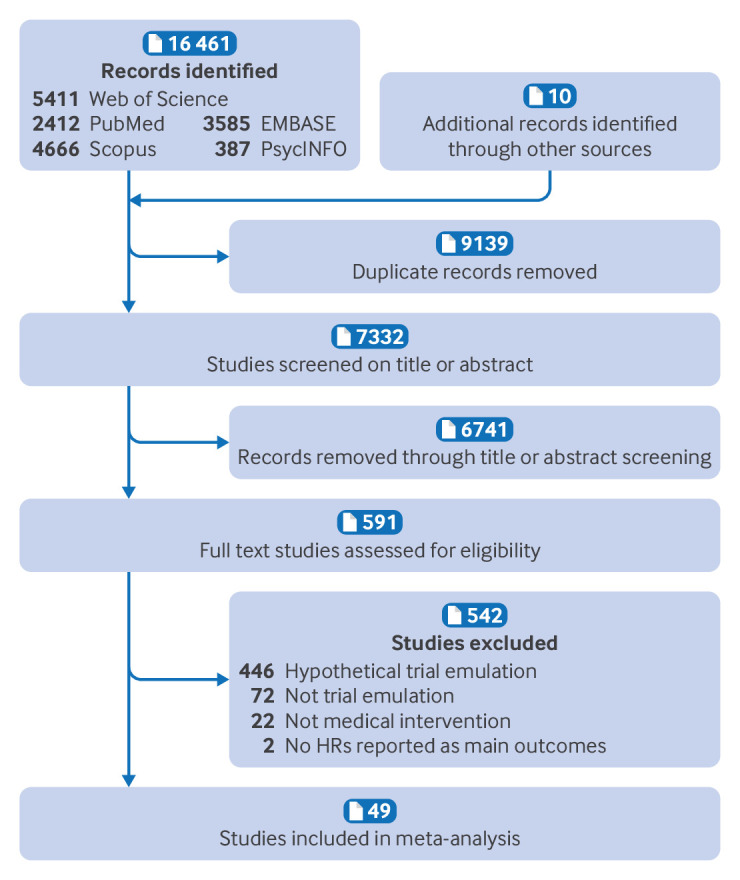
Study flow diagram. HR=hazard ratio

After addition of records identified through other sources and removal of duplicates, 7332 unique records were retrieved, of which we included 49 (with 106 target trial emulation-randomised controlled trial pairs)^5^ ^11^ ^22^ ^23^ ^24^ ^25^ ^26^ ^27^ ^28^ ^29^ ^30^ ^31^ ^32^ ^33^ ^34^ ^35^ ^36^ ^37^ ^38^ ^39^ ^40^ ^41^ ^42^ ^43^ ^44^ ^45^ ^46^ ^47^ ^48^ ^49^ ^50^ ^51^ ^52^ ^53^ ^54^ ^55^ ^56^ ^57^ ^58^ ^59^ ^60^ ^61^ ^62^ ^63^ ^64^ ^65^ ^66^ ^67^ ^68^ (fig 1). At full text review, most exclusions (446/541; 82%) were for hypothetical trial emulations.

The vast majority (99; 93%) of the 106 target trial emulation-randomised controlled trial pairs included examined drug interventions, with more than half in cardiology (43; 41%) or oncology (19; 18%) (table 2). Mortality and major adverse cardiovascular events were the most commonly assessed outcomes ( both 29; 27%). The data source was claims data in half of the pairs (50; 47%). In more than half of target trial emulations, matching was used to emulate randomisation (53; 50%), with another third using weighting (28; 26%). On average, patients included in target trial emulations (versus randomised controlled trials) were slightly older and slightly more often male. The 49 studies included encompassed a total of 106 target trial emulation-randomised controlled trial pairs. Among these studies, 17 (35%) publicly registered their protocol and 16 (33%) followed published reporting guidelines, either STROBE (n=11), International Society for Pharmacoeconomics and Outcomes Research (ISPOR; n=3), both STROBE and ISPOR (n=1), or Reporting of Studies Conducted Using Observational Routinely Collected Health Data (n=1).

**Table 2:** Characteristics of target trial emulation-randomised controlled trial pairs. Values are numbers (percentages) unless stated otherwise

Characteristic	Value (n=106)
**Type of treatment strategy**	
Drug	99 (93)
Surgical	5 (5)
Medical device	2 (2)
**Domain**	
Cardiology	43 (41)
Oncology	19 (18)
Endocrinology	12 (11)
Neurology	11 (10)
Respiratory	5 (5)
Other*	16 (15)
**Outcome**	
Venous thromboembolism	9 (8)
Major adverse cardiac event	29 (27)
Mortality	29 (27)
Respiratory outcome	5 (5)
Stroke	9 (8)
Other	25 (24)
**Data source**	
Claims data	50 (47)
Linked data	17 (16)
Registry	15 (14)
Electronic medical record/routinely collected data	11 (10)
Prospective cohort	9 (8)
Randomised trial	4 (4)
**Database region**	
US	52 (49)
Non-US	47 (44)
Multiple countries	7 (7)
**Design characteristics**	
Confounding adjustment methods:	
Matching	53 (50)
Weighting	29 (27)
G-method	10 (9)
Doubly robust	12 (11)
Both matching and weighting	2 (2)
Baseline characteristics†:	
Mean (SD) age difference, years	2.52 (4.64)
Median (IQR) age difference, years	2.40 (-0.70 to 5.50)
Mean (SD) sex proportion difference	0.16 (12.20)
Median (IQR) sex proportion difference	0.00 (-6.90 to 4.80)
**“Close emulation” characteristics** 5 8	
Comparator emulation‡:	
Good	75 (71)
Moderate	28 (26)
Poor	3 (3)
Outcome emulation§:	
Good	73 (69)
Moderate	32 (30)
Poor	1 (1)
Mixed effect of randomisation and discontinuation of baseline maintenance medication (no)	96 (91)
Start of follow-up in hospital (no)	91 (86)
Run-in that selects responders to one arm (no)	93 (88)
Delayed effects of intervention (no)	98 (92)

IQR=interquartile range; SD=standard deviation.

* Other domains were nephrology (n=3), gastroenterology (n=2), rheumatology (n=2), intensive care (n=2), orthopaedics (n=2), dermatology (n=2), infectious diseases (n=2), and obstetrics (n=1).

† Positive values indicate that emulation population had higher average age and greater proportion of male patients.

‡ Comparator emulation quality was classified as “good” if randomised controlled trial (RCT) used active comparator and emulation applied same or clinically similar active drug; “moderate” if RCT used placebo and emulation used proxy or inactive comparator; and “poor” if non-equivalent comparator was used.

§ Outcome emulation quality was classified as “good” if outcome was reliably identifiable with high specificity and completeness; “moderate” if outcome had lower specificity or high missingness; and “poor” if outcome could not be directly captured or only weak proxies were available.

According to the “close emulation” criteria of Wang and colleagues,^5^ two thirds of the comparator and outcome emulations were rated as good, with most others rated as moderate and very few as poor (table 2). The vast majority of trials did not mix the effect of randomisation and discontinuation of baseline maintenance therapy, did not start follow-up in hospital, did not have a run-in that selected responders to one treatment arm (and that required a stable standard of care, tolerance of the study drug, or discontinuation of maintenance drugs, which could not be emulated in clinical practice data), and did not have a delayed effect of the intervention (on the basis of cumulative incidence curves). For design and characteristics for individual studies, see supplementary table A.

### Risk of bias

Most target trial emulations were rated as having some concerns (39/49; 80%), owing especially to deviations from intended interventions and residual confounding. A minority (six; 12%) were judged to be at low risk of bias, and four (8%) were at serious risk of bias, owing to serious bias in outcome measurement or from intended interventions^35^ ^52^ ^57^ ^66^ (supplementary figures A and B). More than half of trials (45/80; 56%) were at low risk of bias, whereas the remaining 34 (43%) were rated as having some concerns, primarily owing to open label study designs and lack of blinding of outcome assessment (supplementary figures C and D).

### Target trial emulation-randomised controlled trial concordance

**Fig 2:**
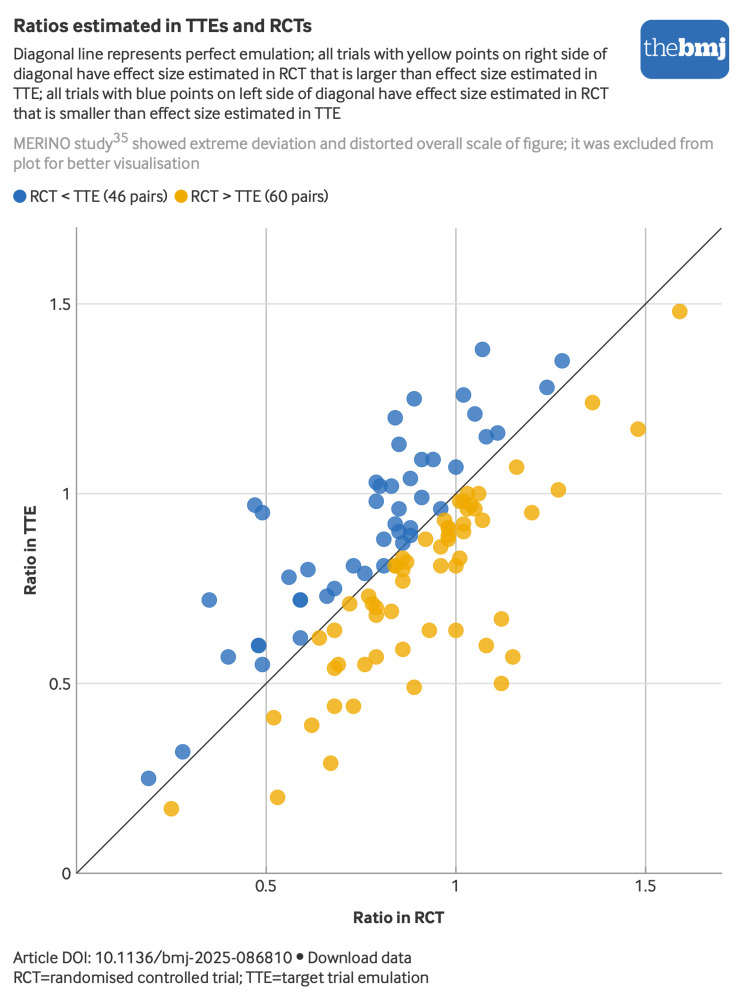
Ratios estimated in target trial emulations and randomised controlled trials. An interactive version of this graphic and downloadable data are available at https://public.flourish.studio/visualisation/29641202/

Most target trial emulation-randomised controlled trial pairs met one or more of the binary agreement metrics for concordance (table 3): standardised difference agreement (84/106; 79%), estimate agreement (73/106; 69%), full agreement for statistical significance (53/106; 50%), and partial agreement for statistical significance (20/41; 49%). The Pearson coefficient between target trial emulation-randomised controlled trial pairs was 0.58 (95% confidence interval (CI) 0.43 to 0.69), and the intraclass correlation coefficient was 0.54 (95% CI 0.39 to 0.66), consistent with moderate correlation. (See supplementary table B for detailed agreement metrics for individual studies and supplementary table C for detailed comparators and outcomes for individual studies). Figure 2 shows that target trial emulation (compared with randomised controlled trial) effect estimates were more often lower (60/106; 57% of pairs; yellow dots below the line with a slope of 1) than higher (44/106; 42% of pairs; blue dots); in 63/106 (59%) of target trial emulation-randomised controlled trial pairs, the percentage deviation of the estimate was below 20%. Of note, the MERINO study (study number 17 in supplementary tables A and B) was an outlier^35^; in this emulation, mortality was assessed at 25 days, rather than at 30 days in the randomised controlled trial, owing to follow-up limitations.

**Table 3:** Agreement metrics between target trial emulations and corresponding randomised controlled trials. Values are numbers (percentages) unless stated otherwise

Metric	All TTE-RCT pairs (n=106)			Study independent TTE-RCT pairs (n=49 pairs)	
	ALL (n=106)	Close emulation (n=62)		ALL (n=49)	Close emulation (n=33)
SDA	84 (79)	54 (87)		43 (88)	31 (94)
EA*	73 (69)	49 (79)		39 (80)	29 (88)
FASS†	53 (50)	36 (58)		25 (51)	17 (52)
PASS‡	20/41 (49)	9/28 (32)		8/11 (73)	5/8 (63)
Pearson (95% CI)	0.58 (0.43 to 0.69)	0.83 (0.73 to 0.89)		0.64 (0.44 to 0.78)	0.87 (0.75 to 0.93)
ICC (95% CI)	0.54 (0.39 to 0.66)	0.81 (0.70 to 0.88)		0.54 (0.31 to 0.71)	0.87 (0.76 to 0.93)

CI=confidence interval; EA=estimate agreement; FASS=full agreement for statistical significance; ICC=intraclass correlation coefficient; PASS=partial agreement for statistical significance; RCT=randomised controlled trial; SDA=standardised difference agreement; TTE=target trial emulation.

* Adjusted TTE study point estimates falling within 95% CI of corresponding RCT result.

† Adjusted TTE study and RCT estimates and CIs on same side of null.

‡ Meets pre-specified non-inferiority criteria even though TTE study may have indicated superiority.

**Fig 3:**
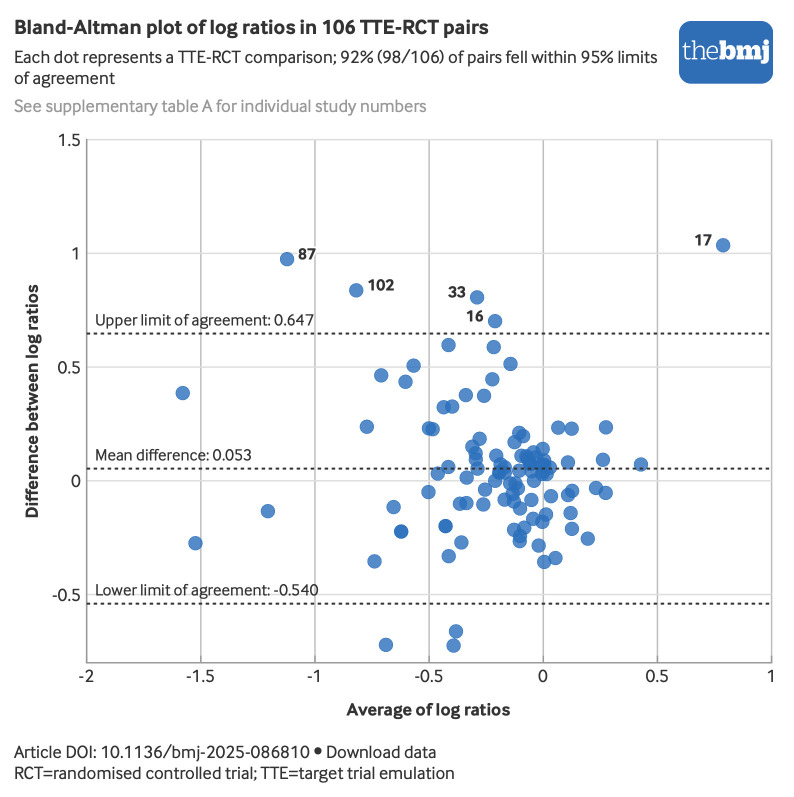
Bland-Altman plot of log ratios in 106 target trial emulation-randomised controlled trial pairs. An interactive version of this graphic and downloadable data are available at https://public.flourish.studio/visualisation/29642371/

Figure 3 presents the Bland-Altman plot, showing no clear trends in target trial emulation-randomised controlled trial differences over the range of average ratio values observed. The overall pooled ratio of ratios was 0.97 (95% CI 0.92 to 1.03), with moderate heterogeneity (I^2^=42%), but 88/106 (83.0%) of target trial emulation-randomised controlled trial pairs fell within the predefined concordance range (ratio of ratios 0.70 to 1.43), suggesting modest variation in concordance.

### Predefined design characteristics

Target trial emulation-randomised controlled trial concordance was greater when Wang’s criteria for “close emulation” (supplementary appendix 2) were met, as observed in 62/106 (58%) target trial emulation-randomised controlled trial pairs. We observed this according to binary metrics (table 3), for all 106 target trial emulation-randomised controlled trial pairs, as well as the 49 study independent pairs (table 3). Also, we saw greater concordance when Wang’s close emulation criteria were met, in univariate regression for differences in log ratios (table 4), and for the specific close emulation criteria of “delayed treatment effect” and good outcome emulation. Additional features that were potentially associated with improved concordance included a difference in sex proportion ≤20% and a smaller difference in participant age (P=0.07 and P=0.09, respectively). In multivariable stepwise regression (supplementary table D), delayed treatment effect, difference in percentage of sex and treatment not started in hospital were retained in the final model. Among these, absence of a delayed treatment effect was significantly associated with target trial emulation-randomised controlled trial concordance; R^2^ was 0.104, indicating that the proportion of variance in target trial emulation-randomised controlled trial concordance explained by the model.

**Table 4:** Linear mixed effects models showing univariate regression coefficients for association between candidate characteristics and differences in log ratios across 106 target trial emulation-randomised controlled trial pairs

Characteristics	Univariate coefficient* (95% CI)	P value	FDR correction	Bonferoni correction
Close emulation (yes)	−0.132 (−0.220 to −0.044)	0.004	0.007	0.094
**Participant characteristics**				
Smaller difference in age	−0.011 (−0.023 to 0.002)	0.09	0.131	1
Smaller difference in sex proportions	−0.004 (−0.009 to 0.001)	0.13	0.166	1
Sex proportion difference ≤20%	−0.136 (−0.281 to 0.010)	0.07	0.102	1
**Intervention characteristics**				
Discontinuation of maintenance (no)	0.000 (−0.145 to 0.145)	1.00	0.999	1
Run-in to one arm (no)	0.061 (−0.073 to 0.196)	0.37	0.420	1
Treatment started in hospital (no)	−0.103 (−0.232 to 0.026)	0.11	0.153	1
Delayed effect (no)	−0.171 (−0.326 to −0.017)	0.03	0.052	0.723
Dose titration (yes)	−0.067 (−0.175 to 0.040)	0.22	0.261	1
**Control characteristics**				
Comparator emulation (good)	−0.025 (−0.122 to 0.073)	0.62	0.674	1
Placebo control (yes)	−0.018 (−0.123 to 0.087)	0.73	0.764	1
**Outcomes**				
Outcome emulation (good)	−0.089 (−0.185 to 0.006)	0.07	0.102	1

CI=confidence interval; FDR=false discovery rate.

* Adjustments by linear mixed effects models, with random intercepts to account for study level clustering and multiple comparison adjustments.

**Fig 4:**
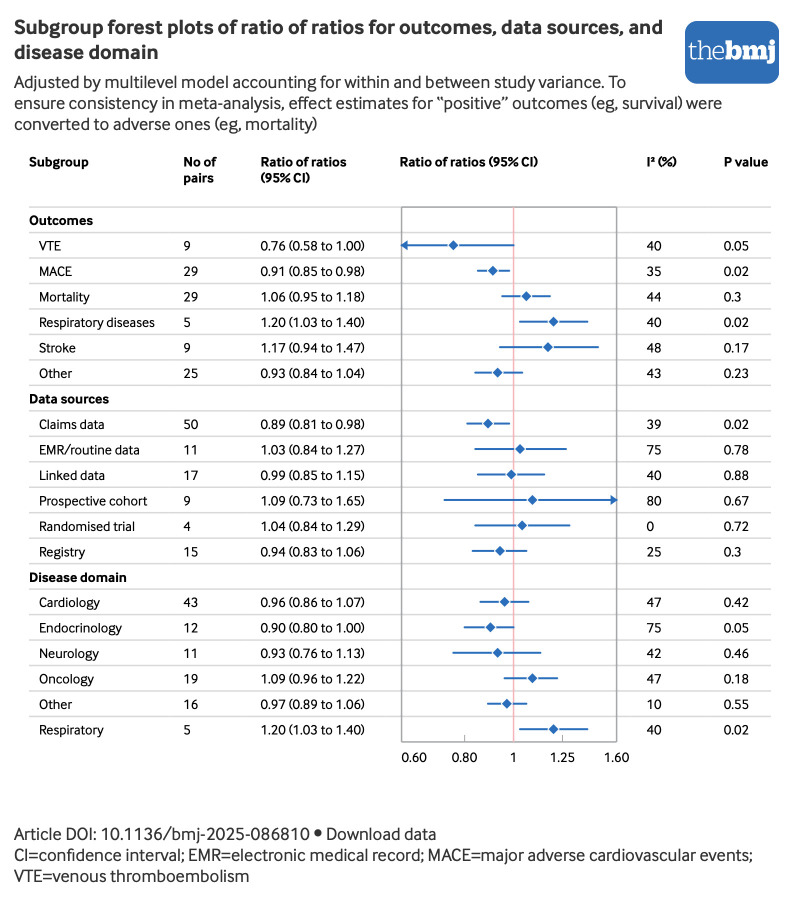
Subgroup forest plots of ratio of ratios for outcomes, data sources, and disease domain. An interactive version of this graphic and downloadable data are available at https://public.flourish.studio/visualisation/29625177/

**Fig 5:**
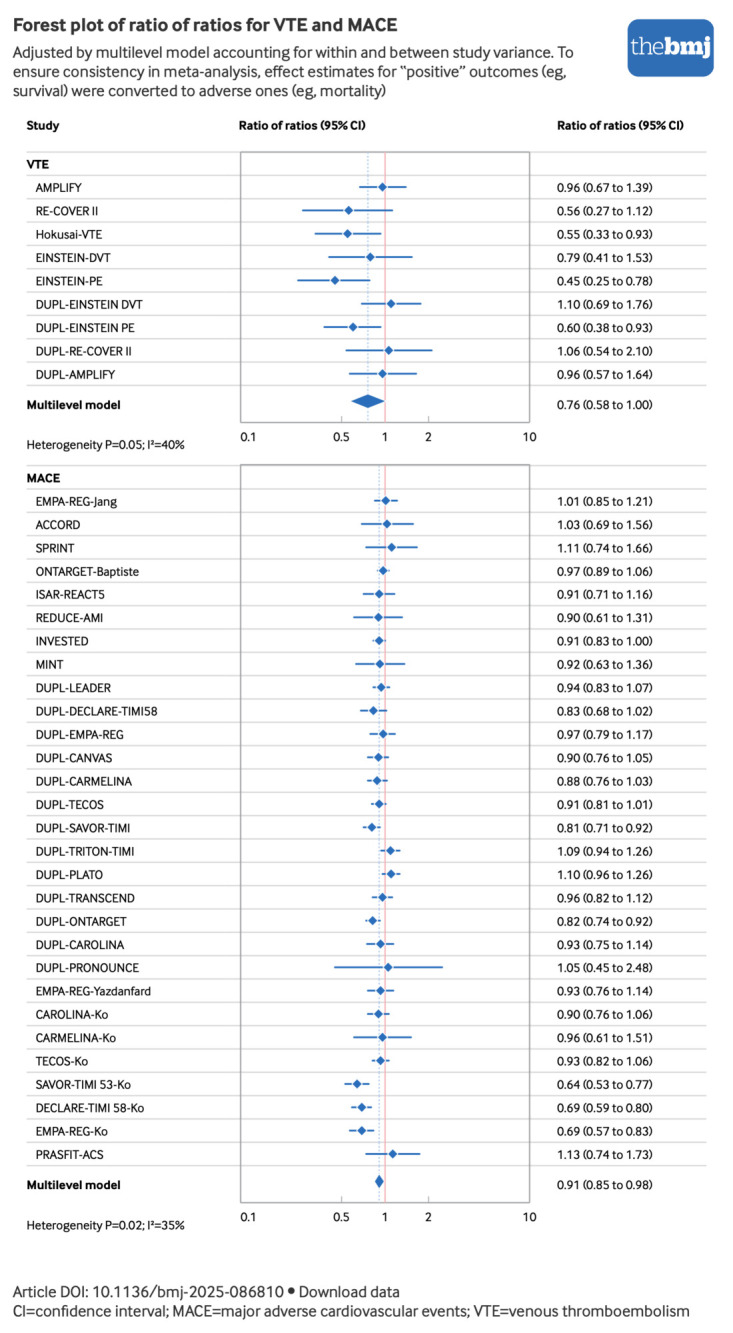
Forest plot of ratio of ratios for venous thromboembolism and major adverse cardiovascular events. An interactive version of this graphic and downloadable data are available at https://public.flourish.studio/visualisation/29628599/

**Fig 6:**
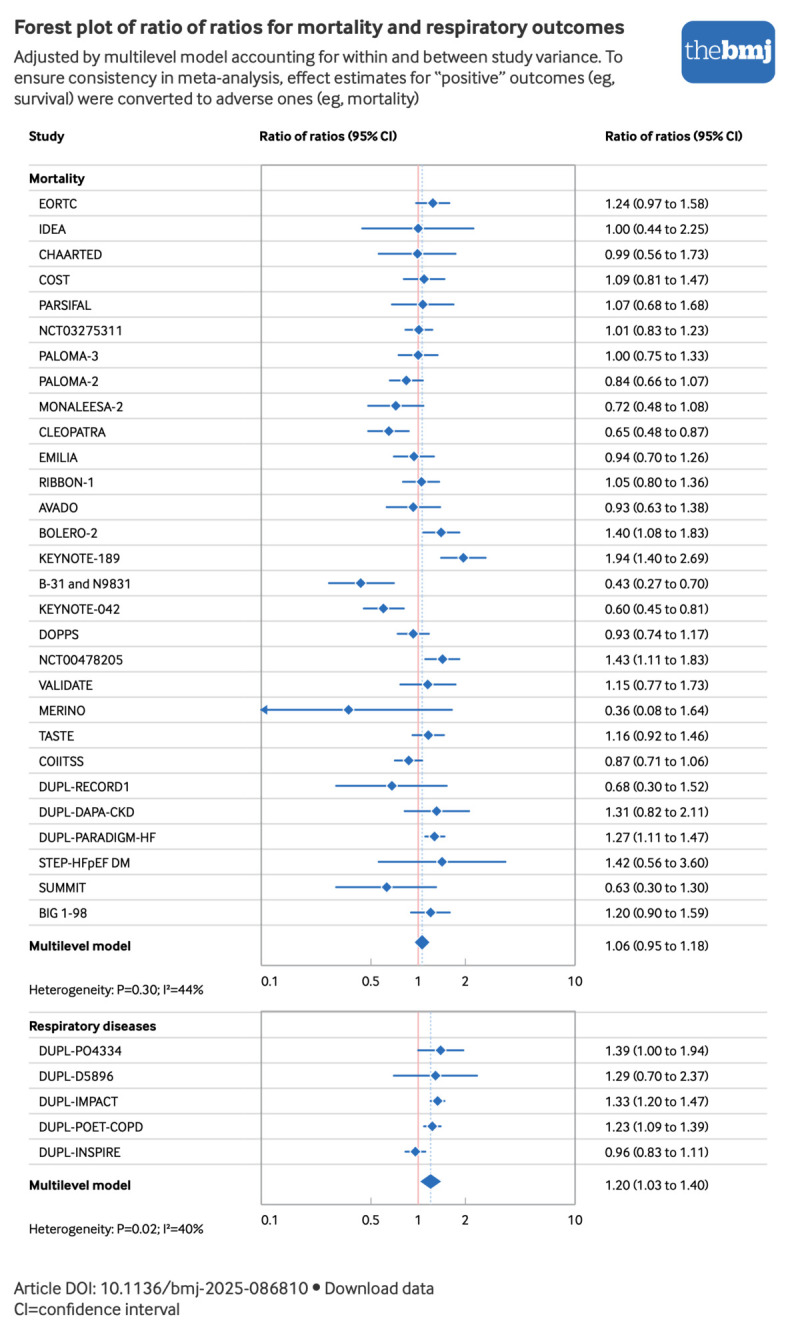
Forest plot of ratio of ratios for mortality and respiratory outcomes. An interactive version of this graphic and downloadable data are available at https://public.flourish.studio/visualisation/29628896/

**Fig 7:**
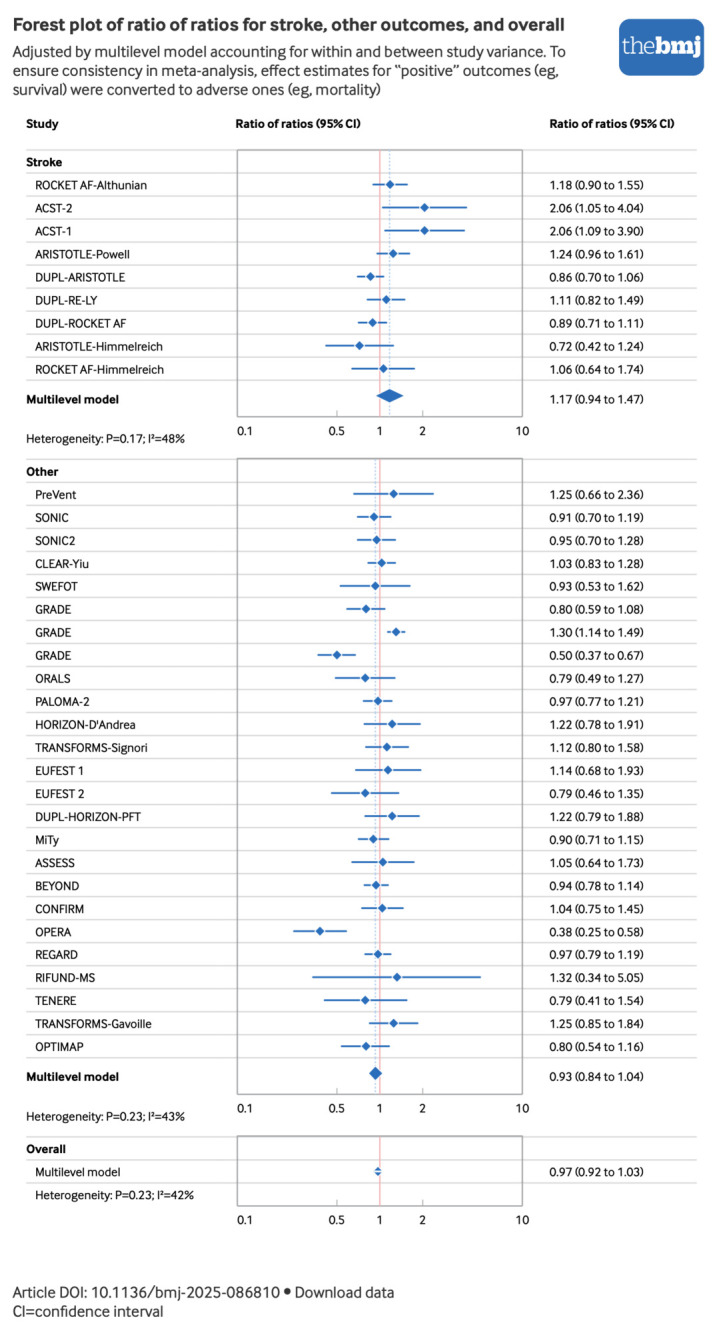
Forest plot of ratio of ratios for stroke, other outcomes, and overall. An interactive version of this graphic and downloadable data are available at https://public.flourish.studio/visualisation/29637710/

In subgroup analyses, compared with randomised controlled trials, target trial emulations consistently underestimated the outcomes of venous thromboembolism (ratio of ratios 0.76, 95% CI 0.58 to 1.00; I^2^=40%; n=9; P=0.05) or major adverse cardiovascular events (0.91, 0.85 to 0.98; I^2^=35%; n=29; P=0.02), but overestimated respiratory outcomes (1.20, 1.03 to 1.40; I^2^=40%; n=5; P=0.02) (fig 4; fig 5; fig 6; fig 7). Target trial emulations based on claims data tended to underestimate the treatment effects compared with randomised controlled trials (ratio of ratios 0.89, 95% CI 0.81 to 0.98; I^2^=39%; n=50; P=0.02) (fig 4). By disease domains, studies involving respiratory conditions showed overestimation, with limited sample size (ratio of ratios 1.20, 1.03 to 1.40; I^2^=40%; n=5; P=0.02) (fig 4).

For intervention type (drugs, surgical procedures, and medical devices), only the studies involving medical devices were concordant with low heterogeneity, although the sample size was limited (supplementary figure E). Studies using multicountry databases showed high concordance, with limited sample size; studies based solely on US databases or non-US databases showed moderate heterogeneity (supplementary figure F). Regarding confounding adjustment methods, both the matching method and the doubly robust method showed good concordance with moderate heterogeneity (supplementary figure G). With respect to effect metrics, risk ratio based analyses and hazard ratio based analyses yielded comparable results (supplementary figure H).

The following factors were not associated with greater target trial emulation-randomised controlled trial concordance, and heterogeneity remained moderate to substantial: estimand (average treatment effect and average treatment effect on treated) categories (supplementary figure I), reporting the use of any guidelines (supplementary figure J), protocol registration (supplementary figure K), the corresponding trial design (non-inferiority versus superiority) (supplementary figure L), and design characteristics (supplementary figure M).

### Sensitivity analyses

Sensitivity analyses yielded robust results across all scenarios. Post hoc exploration with leave-one-out cross validation (supplementary figure N) across 49 studies and 106 target trial emulation-randomised controlled trial pairs, suggested that the overall Pearson correlation coefficient remained stable when individual studies were sequentially removed. The exception was leaving out the MERINO study,^35^ which significantly enhanced the correlation coefficient.

The pooled ratio of ratios varied little from 0.95 to 1.01, and all corresponding 95% confidence intervals included the null value of 1.0 (table 5). Heterogeneity varied from 5% to 54%. Among the subset of study independent target trial emulation-randomised controlled trial pairs with minimal standardised difference, pooled ratio of ratios estimates remained consistent (0.99 to 1.01) with substantially lower heterogeneity (I^2^=5-23%), suggesting greater internal consistency within study independent target trial emulation-randomised controlled trial pairs.

**Table 5:** Sensitivity analyses of ratio of ratios (RoR) for concordance between target trial emulations and randomised controlled trials

Sensitivity analysis	No of pairs	Pooled RoR (95% CI)	I^2^
**All TTE-RCT pairs**	106	0.97 (0.92 to 1.03)	42
Exclude outlier (No 17)	105	0.97 (0.92 to 1.03)	45
Exclude TTE using randomised trial datasets (No 1,26,27,48)	102	0.97 (0.91 to 1.03)	43
Exclude pairs with high risk of bias (No 17,46,51,52,103)	101	0.97 (0.92 to 1.03)	41
Exclude pairs from DUPLICATE-RCT (No53-81)	77	0.97 (0.91 to 1.04)	45
Exclude pairs with sex proportion differences exceeding 20% (No 7,8,30,47,71,77-79,103)	97	0.97 (0.91 to 1.02)	40
Exclude TTE reporting risk ratio	81	0.95 (0.88 to 1.02)	43
**Study independent minimum SD TTE-RCT pairs**	49	1.01 (0.95 to 1.07)	20
Exclude outlier (No 17)	48	1.01 (0.95 to 1.08)	23
Exclude TTE using randomised trial datasets (No 1,27,48)	46	1.01 (0.95 to 1.08)	22
Exclude pairs with high risk of bias (No 17,46,51,103)	46	1.00 (0.95 to 1.04)	5
Exclude pairs with sex proportion differences exceeding 20% (7,8,47,103)	45	1.01 (0.95 to 1.07)	19
Exclude TTE reporting risk ratio	31	0.99 (0.92 to 1.06)	12
**Study independent maximum SD TTE-RCT pairs**	49	0.98 (0.91 to 1.06)	48
Exclude outlier (No 17)	48	0.98 (0.91 to 1.06)	54
Exclude TTE using randomised trial datasets (No 1,28,48)	46	0.98 (0.90 to 1.06)	50
Exclude pairs with high risk of bias (No 17,46,52,103)	45	0.99 (0.92 to 1.06)	45
Exclude pairs with sex proportion differences exceeding 20% (No 7,8,47,77,103)	44	0.96 (0.89 to 1.04)	46
Exclude TTE reporting risk ratio	31	0.96 (0.88 to 1.06)	39

See supplementary table A for individual study numbers.

RCT=randomised control trial; SD=standardised difference; TTE=target trial emulation.

## Discussion

In this systematic review of 106 target trial emulation-randomised controlled trial pairs for health interventions, we found no significant systematic difference overall in treatment effect estimates between target trial emulations and their corresponding trials. However, substantial between pair heterogeneity and several notable discrepancies warrant cautious interpretation and underscore the potential influence of data source, nature of the outcome, and emulation design differences with randomised controlled trials.

### Features associated with concordance

Within our 106 target trial emulation-randomised controlled trial pairs, we found an overall ratio of ratios of 0.97 (95% CI 0.92 to 1.03; I^2^=42%), with moderate overall concordance (Pearson’s r=0.58). Of 21 predefined features, we identified several key features that were significantly associated with target trial emulation-randomised controlled trial concordance, giving greater confidence in results of target trial emulations. Data architecture mattered; emulations using linked data, registries, randomised trial datasets, and multicountry database showed higher concordance than those based on standalone claims/electronic health record data. Delayed effect was linked to weaker alignment of results. The effects of interventions on outcomes of venous thromboembolism and major adverse cardiovascular events were typically underestimated, whereas those on respiratory outcomes were overestimated, likely owing to lower specificity, greater missingness in observational data, or time varying treatment effects, with longer follow-up and higher adherence in trials contrasted with shorter treatment durations in routine clinical practice.^69^ Together, these results delineate conditions for closer alignment and highlight actionable levers: target population matching, precise outcome emulation, and richer data linkage.

### Strengths and limitations of study

To our knowledge, this is the first systematic review and meta-analysis evaluating concordance between target trial emulations and their corresponding randomised controlled trials.^7^ ^70^ Our work corroborates and generalises the key conclusions from RCT-DUPLICATE,^8^ ^70^ extending them through broader coverage, multilevel pooling, and a more detailed mapping of design elements that promote alignment across a wider range of studies and independent datasets. Moreover, of 21 predefined emulation design features, we identified several key characteristics that significantly influenced target trial emulation-randomised controlled trial concordance. This provides actionable methodological insights for improving the design, implementation, and interpretation of future benchmarking target trial emulations. Lastly, by reporting eight concordance metrics and doing subgroup and sensitivity analyses, we offer a robust and multidimensional assessment of agreement. These findings complement the TARGET framework,^71^ particularly for target trial emulation studies that emulate or benchmark existing randomised controlled trials, by identifying pre-specified design features associated with target trial emulation-randomised controlled trial concordance.

We acknowledge several limitations of our work. Firstly, our assessment of “emulation quality” relied on predefined criteria and the information reported in each study, so some relevant biases may not have been captured by our checklist. Secondly, despite use of multilevel modelling, residual confounding may persist owing to specific factors (for example, overlapping database and trial) that may systematically influence outcome ascertainment. Thirdly, we excluded two eligible emulation studies that reported only continuous outcomes,^72^ ^73^ as their effect measures were not directly comparable with ratio based metrics. Fourthly, owing to the distribution and limited size of the available sample, we were unable to do multilevel subgroup analyses across multiple emulation features. Future analyses with larger and more diverse datasets will allow for more granular exploration of effect modifiers. Lastly, selective reporting is an important consideration in benchmarking studies. In this review, only 35% (17/49) of target trial emulation studies reported a publicly accessible protocol or registration; although this may improve transparency, it is not directly analogous to trial registration and cannot rule out selective non-publication, particularly as all included benchmarking emulations were conducted after publication of the index randomised controlled trial.

### Comparison with other studies

Our findings both echo and extend observations from previous research comparing observational and randomised trial evidence, which have reported mixed results regarding their concordance. For example, a comprehensive review by Anglemyer and colleagues found only a small difference between effect estimates from well conducted observational studies and those from randomised controlled trials (pooled ratio of ratios 1.08, 95% CI 0.96 to 1.22).^12^ In that analysis, most comparisons (23/34; 68%) showed effects that were directionally consistent and of similar magnitude.^12^ Similarly, a recent study focusing on covid-19 treatments found that 78% of observational studies were in agreement with meta-analyses of randomised controlled trials on the same questions.^74^ Our findings of 79% standardised difference agreement, 69% estimate agreement, and moderate correlation align with the aforementioned literature. However, Hemkens and colleagues specifically examined observational studies using routinely collected health data and published before their corresponding trials and found that observational studies tended to markedly overestimate treatment effects (16 comparisons; ratio of ratios for mortality 1.31, 95% CI 1.03 to 1.65; I^2^=0%).^18^ These meta-epidemiological studies provide broad comparisons across heterogeneous observational designs, in which key trial defining elements (for example, the estimand, treatment strategies, and time zero) are often implicit or variably implemented. Such misalignment can introduce immortal time bias, depletion of susceptibles, and misclassification, sometimes exaggerating or even reversing effects relative to trials; one review reported immortal time bias in 57% of observational studies and depletion of susceptibles in 44%.^75^ By way of example, a Swedish registry study that explicitly emulated the IDEAL trial yielded mortality hazard ratios of 0.96 to 0.97 (consistent with IDEAL), whereas traditional analyses that introduced time related/survivor biases reversed the association.^25^ This motivates our focus on explicitly reported target trial emulations, a protocolised approach that pre-specifies the estimand and mirrors key trial elements for randomised controlled trial benchmarking.

Studies involving direct comparative analyses between trial emulations and randomised controlled trials remain scarce. Our results are broadly in line with the RCT-DUPLICATE project, which is one of the few efforts to formally emulate multiple trials and compare results. Wang and colleagues used three claims databases to conduct 32 trial emulations, reporting a strong correlation (Pearson’s r=0.82).^5^ In a post hoc analysis restricted to 16 randomised controlled trials with closer emulation to trial design and measurement criteria, the concordance was even higher (Pearson’s r=0.93). These results are remarkably similar to the pattern we observed: high concordance in the close emulation subgroup and much weaker alignment when critical trial features were not or could not be emulated. The lower overall correlation in our review (Pearson r=0.58 versus 0.82) can be explained by our broader inclusion of diverse clinical areas and data sources. Encouragingly, despite the heterogeneity, we still found that a sizeable portion of target trial emulations reached similar conclusions to randomised controlled trials, and we reinforce RCT-DUPLICATE’s conclusion that fidelity to the target trial protocol is a key determinant of success. Our findings also partially align with the recent secondary analysis by Heyard and colleagues of the RCT-DUPLICATE data.^8^ In that meta-regression of 29 trial emulations, three differences in trial design emerged as significant predictors of target trial emulation-randomised controlled trial discrepancies: interventions initiated during hospital admission, discontinuation of certain baseline treatments at randomisation (which cannot be mimicked in routine care), and delayed onset of the treatment’s effect. We likewise observed that hospital initiation was retained in the final multivariable model of predictors of close emulation, and our composite “close emulation” definition implicitly captured problems of treatment discontinuation and latency of effects. One point of contrast is that we did not observe discontinuation of baseline therapy as a significant standalone factor in our dataset. This difference may be due to the diversity of data sources and clinical contexts in our review. This convergence of evidence strengthens the case that, when the goal is to compare an emulation with an existing randomised controlled trial, observed discrepancies are primarily driven by misalignment in trial defining elements, rather than an inherent inability of observational analyses to be valid.

Several methodological challenges should be considered when designing target trial emulations intended to estimate the same effect, in the same population as the index randomised trial, using observational data. Firstly, careful attention is needed to the eligibility criteria and the target population.^76^ In benchmarking, the ideal contrast is an emulation and an index randomised controlled trial that target the same causal estimand and the same target population. Discrepancies in who is studied, such as if the observational data include older or sicker patients or a different sex distribution than the trial, can lead to effect modification or residual confounding that distorts the comparison between the emulation and the corresponding trial. In our analysis, greater differences in age and sex distributions between the emulated cohort and the corresponding randomised controlled trial were associated with lower concordance of effect estimates. Notably, in two studies that used data from the US National Department of Veterans Affairs and Medicare for emulation,^27^ ^28^ the proportion of male patients in the emulated cohort was significantly higher than in the original randomised controlled trial (male proportion: 98.8% in target trial emulation versus 66% in randomised controlled trial), resulting in poor emulation performance (standardised difference=2.28 and 2.17; both >1.96). This implies that if a benchmarking emulation cannot restrict or adjust to closely match the trial’s population, its results may not replicate the trial’s findings. Investigators should therefore explicitly compare baseline characteristics and, where possible, reweight or trim populations to improve compatibility with the target trial criteria.^55^ In practice, differences in measurement/coding, missingness, and limited covariate overlap may constrain “apples-with-apples” comparisons, even after reweighting; transparent code lists, harmonisation of definitions, and reporting of balance before and after adjustment are needed to contextualise any residual differences. By contrast, population extension emulations intentionally target different populations. In such studies, different eligibility is by design, not a flaw. Such discordance may be explained by differences in the target population or residual bias in the observational study.

Secondly, treatment initiation and timing are critical. We found that scenarios in which the randomised controlled trial treatment was initiated in hospital (such as immediately after surgery or during an acute event) were prone to discordant results when emulated. A likely reason is that observational databases (for example, insurance claims) often do not capture inpatient drug use or exact timing of initiation.^5^ The emulation might observe treatment only from the point of discharge, effectively missing early treatment exposures and introducing immortal time or selection biases.^77^ Defining time zero in alignment with the trial is a known challenge in target trial emulation studies.^78^ Potential solutions include linking multiple data sources (for instance, merging claims with hospital electronic health records to capture inpatient treatments) or focusing target trial emulation analyses on interventions initiated in outpatient settings where timing can be aligned more reliably.^79^

Thirdly, maintaining fidelity in treatment strategies (including exposure definitions), comparator conditions, and outcome definitions is essential. Randomised controlled trials often use a placebo or a very specific comparator condition, and they rigorously define outcomes. By contrast, emulations must approximate these conditions using routinely collected data, for which the comparability and validity of outcome definitions may be limited. In our analysis, lower quality of outcome emulation—for instance, outcomes measured with low clinical specificity or high levels of missingness (for example, laboratory test results such as glycated haemoglobin, administrative definitions of venous thromboembolism, or less well validated composite endpoints)—was associated with greater discrepancies between target trial emulation and randomised controlled trial results. This finding aligns with previous concerns that misclassification bias is more likely for outcomes that are not routinely or reliably captured in observational datasets,^18^ ^80^ such as thrombotic events confirmed by imaging or adjudicated cardiovascular endpoints. When such alignment is not feasible, explicitly acknowledging this limitation and doing sensitivity analyses to strengthen the robustness of findings is essential.^80^ This could include outcome misclassification simulations, restriction to subsets with more complete data, use of external validation datasets for calibration, and assessing the completeness and accuracy assessment of outcome capture within a given database (for example, by comparing registry linkage or assessing loss to follow-up). Database type is also critical. Registry databases typically provide prospectively defined outcomes, standardised and audited data collection, more complete recording of key confounders, and longitudinal linkage (for example, to mortality files),^81^ thereby improving outcome ascertainment and reducing misclassification compared with standalone claims or electronic health record data. Compared with registry or linked data sources, studies based solely on claims data are more prone to bias owing to limited clinical detail, potential exposure and outcome misclassification, and residual confounding.^79^ ^82^

### Policy implications

The ability of target trial emulations to faithfully reproduce results of randomised controlled trials has important implications for evidence based decision making. Our results provide cautious optimism about the role of observational evidence and also highlight that target trial emulations are most useful when close emulations can be achieved. Well conducted randomised controlled trials remain the gold standard for establishing causal treatment effects.

Consensus based reporting guidance has just been published for observational studies explicitly emulating a target trial—the TARGET (Transparent Reporting of studies Emulating a Target Trial) statement.^71^ Only 16/49 (33%) studies in our review and 22% in Hansford and colleagues’ reported using any guidance.^83^ Future methodological work should quantify how adherence to checklist items relates to reporting consistency and reproducibility and inform implementation and potential extensions of the guideline. Similarly, only 35% of target trial emulation studies are currently registered; pre-registering target trial emulation protocols—akin to trial registration—would enhance transparency.

### Conclusions

Current target trial emulations achieve moderate concordance when replicating corresponding target randomised controlled trials. Enhancing emulation design is associated with improved target trial emulation-randomised controlled trial concordance, such as through better baseline and outcome emulation and the use of multisource linked databases.

## What is already known on this topic

An emulation of 32 highly selected randomised controlled trials (RCTs) using three US healthcare claims databases found strong concordance between the results
A secondary analysis of these emulations identified three key design related factors as major sources of discordance
How well TTEs reproduce the effects observed in their reference RCTs remains unclear

## What this study adds

This systematic review and meta-analysis evaluating concordance across a broad sample of 106 TTE-RCT pairs found moderate concordance of TTEs with their corresponding RCTs
Concordance could be improved by closer emulation design, including better baseline and outcome emulation, and leveraging multisource linked databases

## Data Availability

The analytical dataset can be made available on reasonable request, subject to a research protocol and collaboration agreement. The R code used for statistical analyses and figure generation is provided in the supplementary materials (appendix 3).

## Web extra

Extra material supplied by authors

Web appendix: Supplementary materials

Web appendix: Original uncorrected article PDF

## Notes

Contributors: LAM and YW contributed equally as joint senior authors. CW conceived the study, did literature screening and data extraction, cleaned and analysed the data, and drafted and revised the manuscript. DT did literature screening and data extraction, and drafted and revised the manuscript. PvD supervised the work and revised the manuscript. CJ advised on and implemented additional sensitivity analyses, and drafted and revised the manuscript. LL cleaned and analysed the data, and drafted and revised the manuscript. YW conceived the study, developed the statistical analysis plan, supervised the work, revised the manuscript, and shares senior authorship. LAM conceived the study, supervised the work, drafted and revised the manuscript, shares senior authorship, and is the guarantor. The corresponding author attests that all listed authors meet authorship criteria and that no others meeting the criteria have been omitted.

Transparency: The lead author (the manuscript’s guarantor) affirms that the manuscript is an honest, accurate, and transparent account of the study being reported; that no important aspects of the study have been omitted; and that any discrepancies from the study as planned (and, if relevant, registered) have been explained.

Dissemination to participants and related patient and public communities: As this study was based on secondary analysis of published data, there were no direct participants to whom results could be disseminated. Findings will be disseminated through publication and academic presentations.

Provenance and peer review: Not commissioned; externally peer reviewed.
