# Concordance between target trial emulation and randomised controlled trials: systematic review and meta-analysis

**DOI:** 10.1136/bmj-2026-100303

**Published:** 2026-07-22

In this paper by Wang and colleagues (BMJ 2026;393:e086810; doi:10.1136/bmj-2025-086810), errors in the supplementary tables and extraction dataset were identified after publication.

The authors identified an inconsistency between supplementary table A and table 3 regarding the number of “close emulation” target trial emulation-randomised controlled trial pairs. Following review of the source data and analysis files, the supplementary table was found to contain an incorrect classification, whereas the analysis dataset used for table 3 was correct.

Also, the authors identified additional data extraction or transcription errors in supplementary table B affecting effect estimates or confidence intervals for individual target trial emulation-randomised controlled trial pairs. These included errors in confidence intervals and use of effect estimates corresponding to different endpoints. One included comparison (No 50, RESONATE-2) was also excluded after eligibility reassessment; this comparison had partially relied on randomised controlled trial data, rather than providing a directly benchmarked, emulation-randomised controlled trial comparison.

The dataset was corrected and the analyses were re-run. The corrected results led to small numerical changes, including: summary Pearson correlation coefficient, originally 0.59 (95% confidence interval 0.45 to 0.70), updated to 0.58 (0.43 to 0.69); summary ratio of ratios, originally 0.96 (0.92 to 1.01; I^2^=36%), updated to 0.97 (0.92 to 1.03; I^2^=42%). In univariate analyses exploring variables associated with close emulation, “treatment initiated in hospital” was no longer significant, and delayed treatment effect was, but both were and remain in the multivariable model. The main conclusions are unchanged.

The article has been corrected, and the supplementary appendix has been replaced. The original uncorrected PDF of the article is retained as a supplementary file.

