# Supplementary material for: Concordance between target trial emulation and randomised controlled trials: systematic review and meta-analysis
Source: BMJ. 2026 May 19;393:e086810. doi: 10.1136/bmj-2025-086810 (PMC13184834; doi:10.1136/bmj-2025-086810)
Supplement: Supplementary file 1 — Web appendix: Supplementary materials [file waca086810.ww1.pdf]

Supplementary materials

Title: Concordance Between Target Trial Emulation and Randomized Controlled Trials: a systematic review and meta-analysis

Appendix number	Title	Page number
A	Search strategy	2
B	Criteria of close emulation	3
C	Model structure for multilevel meta-analysis	5
**Tables**		
	Table A. Study design and other characteristics	6
	Table B. Effect estimates and agreement metrics	11
	Table C. Comparator, and outcome in TTE-RCT pairs	17
	Table D. Multivariable regression coefficients (95% confidence intervals) for the association between candidate characteristics and differences in log ratios across 106 TTE-RCT pairs	26
**Figures**		
	Figure A. Summary of the bias of the TTE with ROBIN-I tool	27
	Figure B. Summary of the bias of the TTE with ROBIN-I tool	27
	Figure C. Summary of the bias of the RCT with RoB 2 tool	28
	Figure D. Summary of the bias of the RCT with RoB 2 tool	28
	Figure E. Forest plots of the ratio of ratios categorised by interventions	29
	Figure F. Forest plots of the ratio of ratios categorised by database region	29
	Figure G. Forest plots of the ratio of ratios categorised by confounding adjustment methods	30
	Figure H Forest plots of the ratio of ratios categorised by report metrics	30
	Figure I. Forest plots of the ratio of ratios categorised by estimand	31
	Figure J. Forest plots of the ratio of ratios categorised by guideline report	31
	Figure K. Forest plots of the ratio of ratios categorised by protocol registration	32
	Figure L. Forest plots of the ratio of ratios categorised by corresponding trial design (non-inferior vs superiority)	32
	Figure M. Forest plots of the ratio of ratios categorised by emulation design	33
	Figure N. Sensitivity analysis. (A) Sensitivity analysis of leave-one-out cross validation for 106 pairs; (B) Sensitivity analysis of leave-one-out cross validation for 49 studies	34

Appendix A Search strategy

Date of the first search: 01 January 2010 - 01 October 2025

PubMed
#1. emulat*[Title/Abstract]
#2. "target trial"[Title/Abstract]
#3. (randomized controlled trial[Publication Type] OR controlled clinical trial[Publication Type] OR randomized[Title/Abstract] OR placebo[Title/Abstract] OR drug therapy[MeSH Terms] OR randomly[Title/Abstract] OR trial[Title/Abstract] OR groups[Title/Abstract]) NOT (animals[MeSH Terms] NOT humans[MeSH Terms])
#4. (#1 OR #2) AND #3

Embase
#1. emulat*:ti,ab OR "target trial":ti,ab
#2. 'randomized controlled trial'/exp OR 'controlled clinical trial'/exp OR randomized:ti,ab OR placebo:ti,ab OR 'drug therapy'/exp OR randomly:ti,ab OR trial:ti,ab OR groups:ti,ab
#3. #1 AND #2
#4. #3 NOT ('animal'/exp NOT 'human'/exp)

Web of Science
#1: TS=(emulat* OR "target trial")
#2: TS=("randomized controlled trial" OR "controlled clinical trial" OR randomized OR placebo OR "drug therapy" OR randomly OR trial OR groups)
#3: #1 AND #2
#4: #3 NOT TS=("animal" NOT "human")

Scopus
#1. TITLE-ABS-KEY(emulat* OR "target trial")
#2. TITLE-ABS-KEY("randomized controlled trial" OR "controlled clinical trial" OR randomized OR placebo OR "drug therapy" OR randomly OR trial OR groups)
#3. #1 AND #2
#4. #3 AND NOT INDEXTERMS("animal") AND NOT INDEXTERMS("human")

PsycINFO
#1. ab(emulat* OR "target trial") OR ti(emulat* OR "target trial")
#2. ab("randomized controlled trial" OR "controlled clinical trial" OR randomized OR placebo OR "drug therapy" OR randomly OR trial OR groups) OR ti("randomized controlled trial" OR "controlled clinical trial" OR randomized OR placebo OR "drug therapy" OR randomly OR trial OR groups)
#3. #1 AND #2
#4. #3 NOT ((animals AND NOT humans) OR exp animals/)

Appendix B. Criteria of close emulation

These criteria were adapted from Wang et al. (Wang SV, Schneeweiss S, RCT-DUPLICATE Initiative. Emulation of Randomized Clinical Trials With Nonrandomized Database Analyses: Results of 32 Clinical Trials. JAMA.). Operationally, close emulation of trial design was defined as: comparator and outcome emulation criteria 1,2 rated at least 'moderate', with at least one classified as 'good'; and 'yes' to each of criteria 3-6.

Criteria for emulation characteristics assessment

Domain	Score Level	Operational Criteria	Example
**1.Comparator emulation**	**Good**	The RCT used an **active comparator**, and the RWE emulation used the **same** or **clinically similar** active drug. Confounding is expected to be minimal.	RCT: Drug A vs. Drug B. RWE: New users of A vs. B in similar populations, balanced via matching.
	**Moderate**	The RCT used a **placebo**, and the RWE used an **active comparator with no expected effect on the outcome**, with well-balanced risk factors; or a proxy comparator class.	RCT: Drug vs. placebo; RWE: Drug vs. DPP-4i (assumed neutral for CV outcome), with good confounder control.
	**Poor**	RCT used a placebo; RWE used a **non-equivalent comparator** with expected residual confounding (e.g., due to SES, indication bias).	RWE compares expensive drugs to low-cost therapy used by sicker/poorer patients (e.g., sulfonylureas).
**2.Outcome emulation**	**Good**	Outcome can be identified in claims/EHR data with **high specificity and completeness**.	RCT & RWE both use all-cause mortality, from registry or death records.
	**Moderate**	Outcome measured with **lower specificity** or **high missingness** (e.g., labs like HbA1c, or less validated outcomes).	HbA1c changes were identified through sparse outpatient lab results.
	**Poor**	Outcome cannot be captured or only weak proxies are available.	RCT: Symptom improvement; RWE: Proxy via follow-up visits, which poorly reflects actual symptom change.
**3.No mixing of effects of randomisation and discontinuation of baseline maintenance therapy**	**Yes**	RCT did not require stopping baseline therapy, or RWE restricted inclusion to match such a baseline.	RCT excludes diuretics at baseline; RWE does too.
	**No**	RCT required stopping a therapy (e.g., LABA), but RWE patients **did not stop** and continued	RCT: Stop LABA before randomisation; RWE: New ICS use but continued

Domain	Score Level	Operational Criteria	Example
		overlapping meds, **mixing effects**.	LABA, causing confounding.
**4.No start of follow-up in hospital**	**Yes**	Either the RCT did **not** require in-hospital initiation, or RWE could replicate hospital-based treatment starts.	RCT and RWE both define treatment start during hospitalisation for MI.
	**No**	RCT started treatment **in-hospital**, but RWE lacks inpatient medication data and uses **post-discharge Rx** as start, missing early effects.	RCT: Starts therapy in ER; RWE: Only observes outpatient prescriptions (bias via immortal time).
**5.No selective run-in period**	**Yes**	RCT had **no active run-in**, or RWE approximated run-in (e.g., requiring stable treatment for 3 months prior).	RCT requires 3 months prior stable therapy; RWE applies the same eligibility rule using baseline data.
	**No**	RCT included a **run-in period selecting responders**, which RWE could not emulate. This creates post-baseline selection not replicable.	RCT gives 4-week drug run-in and only randomises responders; RWE cannot identify who would respond.
**6.No delayed treatment effects**	**Yes**	Either RCT had no delayed effects, or RWE had sufficient follow-up **and adherence** to observe long-term outcomes.	RCT: effects seen by 3 months; RWE follow-up = 6+ months, with good persistence.
	**No**	RCT had **long follow-up** and time-varying effects; RWE had **shorter follow-up** or poor adherence, missing delayed effects.	RCT: curves diverge after 12 months; RWE: most patients stop by 6 months.

Abbreviations: TTE: target trial emulation; RCT: randomised controlled trial; RWE: real-world evidence; DPP-4i: dipeptidyl peptidase-4 inhibitor; CV: cardiovascular; SES: socioeconomic status; EHR: electronic health record; HbA1c: haemoglobin A1c; LABA: long-acting beta-agonist; ICS: inhaled corticosteroids; ER: emergency room; Rx: prescription; MI: myocardial infarction.

Appendix C. Model structure for multilevel meta-analysis

In this analysis, we modelled two levels of random effects to account for clustering and non-independence across effect estimates (using the metafor package in R):

(1) Study level - representing variation across primary studies that may contribute multiple TTE-RCT comparison pairs.

(2) Pair level - representing residual heterogeneity across individual TTE-RCT pairs within each study.

Each `study_id` identifies a distinct source publication, while `pair_id` identifies each unique TTE-RCT comparison within that study.

Code illustration (simplified):

```
library(metafor)

res <- rma.mv(yi = log_HR,
           V = SE_log_HR^2,
           random = list(~ 1 | study_id, ~ 1 | pair_id),
           method = "REML")
summary(res)
```

Supplementary Tables

Table A. Study design and other characteristics

No. #	Trial name	Data source	confounding adjustment	Comparator	Outcome	Difference in age	Difference in sex (%)	Run in	Placebo control	Treatment started in hospital	Dose titration	Discontinuation	Delayed effect	Close emulation
1	PreVent[1]	Randomised trial	Matching	Good	Good	0	0.4	No	No	Yes	No	No	No	No
2	ROCKET AF[2]	EMR / Routinely collected data	Matching	Moderate	Good	-2	5.4	No	No	No	Yes	No	No	Yes
3	EORTC[3]	Linked data	Weighting	Good	Good	0	-6.4	No	No	No	No	No	No	Yes
4	IDEA[4]	Linked data	G-method	Moderate	Good	5	3.9	No	No	No	Yes	No	No	Yes
5	DOPPS[5]	Registry	G-method	Good	Good	-12.8	0.9	No	No	No	Yes	No	No	Yes
6	NCT00478205[6]	Prospective Cohort	G-method	Moderate	Good	-3.2	4	No	No	Yes	Yes	No	No	No
7	ACST-2[7]	Linked data	G-method	Moderate	Good	-5.6	-32.8	No	No	Yes	No	No	No	No
8	ACST-1[8]	Linked data	G-method	Good	Moderate	-6.9	-32.8	No	No	Yes	No	No	No	No
9	SONIC[9]	Claims data	Matching	Good	Moderate	-4.2	-4.8	No	No	No	No	No	No	Yes
10	SONIC2[10]	Claims data	Matching	Good	Moderate	-5.8	3	No	No	No	Yes	No	No	Yes
11	VALIDATE[11]	Linked data	Doubly robust	Good	Good	0	1.2	No	No	No	Yes	No	No	Yes
12	CLEAR [12]	Linked data	Both	Good	Moderate	-1	6.5	No	No	No	Yes	No	No	Yes
13	SWEFOT[13]	Linked data	Weighting	Good	Moderate	-2	8.4	No	No	No	Yes	No	No	Yes
14	GRADE[14]	EMR / Routinely collected data	Weighting	Good	Moderate	-4.6	12.4	No	No	No	Yes	No	No	No
15	GRADE	EMR / Routinely collected data	Weighting	Good	Moderate	-4.6	12.4	No	No	No	Yes	No	No	No
16	GRADE	EMR / Routinely collected data	Weighting	Good	Moderate	-4.6	12.4	No	No	No	Yes	No	No	No
17	MERINO[15]	Linked data	Matching	Good	Poor	-3	3.3	No	No	No	No	No	Yes	No
18	CHAARTED[16]	EMR / Routinely collected data	Matching	Good	Good	-2.7	0	No	No	No	No	No	No	Yes
19	COST[17]	Registry	Doubly robust	Good	Good	6	3	No	No	Yes	No	No	No	No

20	EMPA-REG[18]	EMR / Routinely collected data	Matching	Moderate	Good	7.6	12.4	No	Yes	No	No	No	No	Yes
21	ORALS[19]	Linked data	Weighting	Good	Good	7.4	4	No	No	No	No	No	No	Yes
22	TASTE[20]	Linked data	Matching	Good	Good	-0.1	0.2	No	No	No	No	No	No	Yes
23	PARSIFAL[21]	Linked data	Weighting	Good	Good	-5	0	No	No	No	No	No	No	Yes
24	NCT03275311[22]	Registry	Both	Good	Good	-4.9	0	No	No	No	No	No	No	Yes
25	COIITSS[23]	Claims data	Doubly robust	Good	Good	-3.1	10.7	No	No	Yes	No	No	No	No
26	ACCORD[24]	Randomised trial	Weighting	Good	Good	-0.8	5	No	No	No	No	No	No	Yes
27	SPRINT[24]	Randomised trial	Weighting	Good	Good	0.7	7.2	No	No	No	No	No	No	Yes
28	PALOMA-2[25]	EMR / Routinely collected data	Weighting	Moderate	Moderat e	-5	0	No	Yes	No	No	No	No	No
29	AMPLIFY[26]	Claims data	Matching	Good	Moderat e	-11.7	15.9	No	No	Yes	No	No	No	No
30	RE-COVER II[26]	Claims data	Matching	Good	Moderat e	-13.3	20	No	No	Yes	No	No	No	No
31	Hokusai-VTE[26]	Claims data	Matching	Good	Moderat e	-13.1	17.4	No	No	Yes	No	No	No	No
32	EINSTEIN-DVT[26]	Claims data	Matching	Good	Moderat e	-7.5	13.3	No	No	Yes	No	No	No	No
33	EINSTEIN-PE[26]	Claims data	Matching	Good	Moderat e	-11.1	13.6	No	No	Yes	No	No	No	No
34	PALOMA-3[27]	Prospective Cohort	Weighting	Moderate	Good	-2.4	0	No	No	No	No	No	No	Yes
35	PALOMA-2[27]	Prospective Cohort	Weighting	Moderate	Good	-7.2	0	No	No	No	No	No	No	Yes
36	MONALEESA-2[27]	Prospective Cohort	Weighting	Moderate	Good	-6.9	0	No	No	No	No	No	No	Yes
37	CLEOPATRA[27]	Prospective Cohort	Weighting	Moderate	Good	0.8	0	No	No	No	No	No	No	Yes
38	EMILIA[27]	Prospective Cohort	Weighting	Good	Good	-1.2	0	No	No	No	No	No	No	Yes
39	RIBBON-1[27]	Prospective Cohort	Weighting	Moderate	Good	1.5	0	No	No	No	No	No	No	Yes
40	AVADO[27]	Prospective Cohort	Weighting	Moderate	Good	0.1	0	No	No	No	No	No	No	Yes
41	BOLERO-2[27]	Prospective Cohort	Weighting	Moderate	Good	-4	0	No	No	No	No	No	No	Yes
42	ONTARGET[28]	Linked data	Weighting	Moderate	Good	-4.4	18.9	Yes	No	Yes	Yes	Yes	No	No
43	HORIZON[29]	Claims data	Matching	Moderate	Good	-0.9	-	No	Yes	No	No	No	Yes	No

44	ISAR-REACT5[30]	Claims data	Matching	Good	Good	1.4	2.2	No	No	No	No	No	No	Yes
45	REDUCE-AMI[31]	Linked data	G-method	Good	Good	1	-1.2	Yes	No	No	No	No	Yes	No
46	KEYNOTE-189[32]	EMR / Routinely collected data	G-method	Moderate	Good	1.3	9.6	No	Yes	No	No	No	Yes	No
47	INVESTED[33]	Claims data	Matching	Moderate	Good	-14.5	29.6	No	No	No	No	No	No	Yes
48	MINT[34]	Randomised trial	G-method	Moderate	Good	0	-0.1	No	No	Yes	No	No	No	Yes
49	ARISTOTLE[35]	EMR / Routinely collected data	Matching	Good	Moderate	-1	0	No	No	No	No	No	No	Yes
50	TRANSFORMS[36]	Registry	Matching	Good	Good	1.2	4.2	No	No	No	No	No	No	Yes
51	EUFEST 1[37]	EMR / Routinely collected data	Weighting	Moderate	Good	2.4	-16.3	No	No	No	No	No	No	Yes
52	EUFEST 2[37]	EMR / Routinely collected data	Weighting	Moderate	Good	2.2	-4.3	No	No	No	No	No	No	Yes
53	DUPL-LEADER[38]	Claims data	Matching	Moderate	Good	-3.4	-17.8	Yes	Yes	No	Yes	No	No	Yes
54	DUPL-DECLARE-TIMI58	Claims data	Matching	Moderate	Moderate	1.4	-4.9	Yes	Yes	No	No	No	No	No
55	DUPL-EMPA-REG	Claims data	Matching	Moderate	Good	1.2	-11.9	Yes	Yes	No	No	No	No	Yes
56	DUPL-CANVAS	Claims data	Matching	Moderate	Good	-2	-10.5	Yes	Yes	No	No	No	No	Yes
57	DUPL-CARMELINA	Claims data	Matching	Poor	Good	-6.4	-16.2	No	Yes	No	No	No	No	No
58	DUPL-TECOS	Claims data	Matching	Poor	Moderate	-6.8	-18.1	No	Yes	No	No	No	No	No
59	DUPL-SAVOR-TIMI	Claims data	Matching	Poor	Good	-3.8	-13.7	No	Yes	No	No	No	No	No
60	DUPL-TRITON-TIMI	Claims data	Matching	Good	Good	-3.3	-4.9	No	No	Yes	Yes	No	No	No
61	DUPL-PLATO	Claims data	Matching	Good	Good	-3.3	-4.1	No	No	Yes	Yes	No	No	No
62	DUPL-ARISTOTLE	Claims data	Matching	Good	Good	-6.1	-16.7	No	No	No	No	Yes	No	Yes
63	DUPL-RE-LY	Claims data	Matching	Good	Good	4.7	5.9	No	No	No	No	Yes	No	Yes
64	DUPL-ROCKET AF	Claims data	Matching	Good	Good	4.5	-14.9	No	No	No	No	Yes	No	Yes
65	DUPL-EINSTEIN DVT	Claims data	Matching	Good	Moderate	-14.7	-17	No	No	No	Yes	No	No	Yes
66	DUPL-EINSTEIN PE	Claims data	Matching	Good	Moderate	-8.2	-4.9	No	No	No	Yes	No	No	Yes
67	DUPL-RE-COVER II	Claims data	Matching	Good	Moderate	-7.1	-16.4	No	No	No	No	No	No	Yes

68	DUPL-AMPLIFY	Claims data	Matching	Good	Moderate	-0.6	-10.1	No	No	No	Yes	No	No	Yes
69	DUPL-RECORD1	Claims data	Matching	Good	Good	1	1.6	No	No	No	No	No	No	Yes
70	DUPL-TRANSCEND	Claims data	Matching	Moderate	Good	-4	-14.1	Yes	Yes	No	No	No	No	Yes
71	DUPL-ONTARGET	Claims data	Matching	Good	Good	2.4	-27.2	Yes	No	No	Yes	No	No	No
72	DUPL-HORIZON-PFT	Claims data	Matching	Moderate	Good	-1	0	No	Yes	No	No	No	Yes	No
73	DUPL-DAPA-CKD	Claims data	Matching	Moderate	Moderate	-5.5	-11.4	No	Yes	No	No	No	Yes	No
74	DUPL-PARADIGM-HF	Claims data	Matching	Moderate	Moderate	-4.7	-6.2	No	No	No	No	No	Yes	No
75	DUPL-PO4334	Claims data	Matching	Good	Good	-11.2	1.9	Yes	No	No	No	Yes	No	No
76	DUPL-D5896	Claims data	Matching	Good	Good	-3.3	-1.8	No	No	No	No	Yes	No	No
77	DUPL-IMPACT	Claims data	Matching	Good	Good	-4	-25.5	Yes	No	No	No	Yes	No	No
78	DUPL-POET-COPD	Claims data	Matching	Good	Good	-7.5	-28.3	Yes	No	No	No	Yes	No	No
79	DUPL-INSPIRE	Claims data	Matching	Good	Moderate	-1.5	-44.6	Yes	No	No	No	Yes	No	No
80	DUPL-CAROLINA	Claims data	Matching	Good	Good	-6.3	-12.3	Yes	No	No	Yes	No	No	Yes
81	DUPL-PRONOUNCE	Claims data	Matching	Good	Good	-3	0	No	No	No	Yes	No	No	Yes
82	MiTy[39]	Claims data	Regression	Good	Good	4.2	0	No	Yes	No	No	No	No	Yes
83	EMPA-REG-Yazdanfard[40]	Linked data	doubly robust	Good	Good	-6.5	0	No	Yes	No	No	No	No	Yes
84	ASSESS[41]	Registry	doubly robust	Good	Good	1.9	2.4	No	No	No	No	No	No	No
85	BEYOND	Registry	doubly robust	Good	Moderate	-0.7	6.9	No	No	No	No	No	No	No
86	CONFIRM	Registry	doubly robust	Good	Moderate	1	6.6	No	No	No	No	No	No	No
87	OPERA	Registry	doubly robust	Good	Moderate	1.7	7.8	No	No	No	No	No	No	No
88	REGARD	Registry	doubly robust	Good	Moderate	-0.2	5.4	No	No	No	No	No	No	No
89	RIFUND-MS	Registry	doubly robust	Good	Moderate	0	4.2	No	No	No	No	No	No	No
90	TENERE	Registry	doubly robust	Good	Moderate	-2.4	5.4	No	No	No	No	No	No	No

91	TRANSFORMS-Gavoille	Registry	doubly robust	Good	Moderate	-0.1	8.6	No	No	No	No	No	No	No
92	STEP-HFpEF DM[42]	Claims data	Weighting	Good	Good	-0.3	9	No	Yes	No	No	No	No	Yes
93	SUMMIT	Claims data	Weighting	Good	Good	-5.3	-0.2	No	Yes	No	No	No	No	Yes
94	BIG 1-98[43]	Linked data	Weighting	Good	Good	-7	0	No	No	No	No	No	No	Yes
95	CAROLINA-Ko[44]	Claims data	Matching	Good	Good	1.3	6.9	No	No	No	No	No	No	Yes
96	CARMELINA-Ko	Claims data	Matching	Good	Good	-0.1	5.9	No	Yes	No	No	No	No	Yes
97	TECOS-Ko	Claims data	Matching	Good	Good	-5.7	11.1	No	Yes	No	No	No	No	Yes
98	SAVOR-TIMI 53-Ko	Claims data	Matching	Good	Good	4.8	9.5	No	Yes	No	No	No	No	Yes
99	DECLARE-TIMI 58-Ko	Claims data	Matching	Good	Good	0	-0.3	No	Yes	No	No	No	No	Yes
100	EMPA-REG-Ko	Claims data	Matching	Good	Good	5.1	12.7	No	Yes	No	No	No	No	Yes
101	PRASFIT-ACS[45]	Claims data	Weighting	Good	Good	-0.4	1.4	No	Yes	No	No	No	No	Yes
102	B-31 and N9831[46]	Linked data	G-method	Good	Good	/	0	No	No	No	No	No	No	Yes
103	OPTIMAP[47]	Registry	Weighting	Good	Moderate	3.7	23.5	No	No	No	No	Yes	Yes	No
104	KEYNOTE-042[48]	Linked data	Weighting	Good	Good	-6	18	No	No	No	No	No	No	Yes
105	ARISTOTLE-Himmelreich[49]	Registry	Weighting	Good	Good	-6	12.7	No	No	No	No	No	No	Yes
106	ROCKET AF-Himmelreich	Registry	Weighting	Good	Good	-3	7.6	No	No	No	No	No	No	Yes

Abbreviations: PSM, Propensity score matching; IPW, Inverse probability weighting; EMR, electronic medical record.

Table B. Effect Estimates and Agreement Metrics, ranked by outcomes and standardised differences.

Study No.	Trial name	Outcome	Effect Estimates (95% CI)			Agreement		
			RCT	TTE	SD[d]	SDA	EA	SS
**VTE**								
68	DUPL-AMPLIFY	VTE / VTE Related Death	0.84 (0.60 to 1.18)	0.81 (0.54 to 1.23)	0.13	+	+	+
67	DUPL-RE-COVER II	VTE / VTE Related Death	1.08 (0.64 to 1.80)	1.15 (0.74 to 1.78)	0.18	+	+	+
29	AMPLIFY	VTE / VTE Related Death	0.84 (0.60 to 1.18)	0.81 (0.70 to 0.94)	0.19	+	+	P
65	DUPL-EINSTEIN DVT	VTE	0.68 (0.44 to 1.04)	0.75 (0.62 to 0.90)	0.41	+	+	P
32	EINSTEIN-DVT	VTE	0.68 (0.44 to 1.04)	0.54 (0.33 to 0.89)	0.69	+	+	P
30	RE-COVER II	VTE / VTE Related Death	1.08 (0.64 to 1.80)	0.60 (0.37 to 0.96)	1.64	+		P
31	Hokusai-VTE	VTE / VTE Related Death	0.89 (0.70 to 1.13)	0.49 (0.31 to 0.78)	2.25			P
66	DUPL-EINSTEIN PE	VTE	1.12 (0.75 to 1.68)	0.67 (0.55 to 0.80)	2.26			P
33	EINSTEIN-PE	VTE	1.12 (0.75 to 1.68)	0.50 (0.34 to 0.74)	2.82			P
**MACE**								
81	DUPL-PRONOUNCE	MACE	1.28 (0.59 to 2.79)	1.35 (0.94 to 1.93)	0.12	+	+	+
20	EMPA-REG-Jang	MACE	0.86 (0.74 to 0.99)	0.87 (0.79 to 0.96)	0.13	+	+	+
26	ACCORD	MACE	0.88 (0.73 to 1.06)	0.91 (0.63 to 1.31)	0.16	+	+	
96	CARMELINA-Ko	MACE	1.02 (0.89 to 1.17)	0.98 (0.64 to 1.51)	0.17	+	+	
55	DUPL-EMPA-REG	MACE	0.86 (0.74 to 0.99)	0.83 (0.73 to 0.95)	0.35	+	+	+
48	MINT	MACE	1.16 (1.00 to 1.35)	1.07 (0.74 to 1.51)	0.41	+	+	+
27	SPRINT	MACE	0.73 (0.63 to 0.86)	0.81 (0.56 to 1.18)	0.5	+	+	
101	PRASFIT-ACS	MACE	0.85 (0.62 to 1.16)	0.96 (0.72 to 1.29)	0.56	+	+	
70	DUPL-TRANSCEND	MACE + HHF	0.92 (0.81 to 1.05)	0.88 (0.81 to 0.96)	0.56	+	+	
45	REDUCE-AMI	MACE	0.96 (0.79 to 1.16)	0.86 (0.64 to 1.23)	0.57	+	+	+
80	DUPL-CAROLINA	MACE	0.98 (0.84 to 1.14)	0.91 (0.79 to 1.05)	0.7	+	+	+
42	ONTARGET-Baptiste	MACE	1.01 (0.94 to 1.09)	0.98 (0.94 to 1.02)	0.7	+	+	

			Effect Estimates (95% CI)			Agreement		
Study No.	Trial name	Outcome	RCT	TTE	SD[d]	SDA	EA	SS
83	EMPA-REG-Yazdanfard	MACE	0.86 (0.74 to 0.99)	0.80 (0.70 to 0.92)	0.71	+	+	+
44	ISAR-REACT5	MACE	1.36 (1.09 to 1.70)	1.24 (1.12 to 1.37)	0.74	+	+	+
53	DUPL-LEADER	MACE	0.87 (0.78 to 0.97)	0.82 (0.76 to 0.87)	0.9	+	+	+
60	DUPL-TRITON-TIMI	MACE	0.81 (0.73 to 0.90)	0.88 (0.79 to 0.97)	1.11	+	+	+
97	TECOS-Ko	MACE	0.98 (0.88 to 1.09)	0.91 (0.85 to 0.98)	1.13	+	+	P
95	CAROLINA-Ko	MACE	0.98 (0.84 to 1.14)	0.88 (0.82 to 0.94)	1.26	+	+	P
61	DUPL-PLATO	MACE	0.84 (0.77 to 0.92)	0.92 (0.83 to 1.02)	1.31	+	+	
56	DUPL-CANVAS	MACE	0.86 (0.75 to 0.97)	0.77 (0.70 to 0.85)	1.34	+	+	+
57	DUPL-CARMELINA	MACE	1.02 (0.89 to 1.17)	0.90 (0.84 to 0.96)	1.61	+	+	P
58	DUPL-TECOS	MACE	0.98 (0.88 to 1.09)	0.89 (0.86 to 0.91)	1.71	+	+	P
54	DUPL-DECLARE-TIMI58	HHF + cardiovascular death	0.83 (0.73 to 0.95)	0.69 (0.59 to 0.81)	1.76	+		+
47	INVESTED	MACE	1.05 (0.96 to 1.15)	0.96 (0.95 to 0.98)	1.92	+	+	P
59	DUPL-SAVOR-TIMI	MACE	1.00 (0.89 to 1.12)	0.81 (0.76 to 0.86)	3.16			P
71	DUPL-ONTARGET	MACE + HHF	1.01 (0.94 to 1.09)	0.83 (0.77 to 0.90)	3.58			P
100	EMPA-REG-Ko	MACE	0.86 (0.74 to 0.99)	0.59 (0.52 to 0.67)	3.83			+
99	DECLARE-TIMI 58-Ko	MACE	0.93 (0.84 to 1.03)	0.64 (0.57 to 0.72)	4.72			+
98	SAVOR-TIMI 53-Ko	MACE	1.00 (0.89 to 1.12)	0.64 (0.56 to 0.74)	4.84			
**Mortality**								
4	IDEA	OS	0.96 (0.85 to 1.08)	0.96 (0.43 to 2.14)	0	+	+	+
34	PALOMA-3	OS	0.81 (0.64 to 1.03)	0.81 (0.69 to 0.95)	0	+	+	P
18	CHAARTED	OS	0.72 (0.59 to 0.89)	0.71 (0.42 to 1.19)	0.05	+	+	
24	NCT03275311	OS	0.88 (0.74 to 1.05)	0.89 (0.82 to 0.98)	0.11	+	+	P
23	PARSIFAL	OS	1.00 (0.68 to 1.48)	1.07 (0.86 to 1.35)	0.3	+	+	
39	RIBBON-1	OS	1.11 (0.86 to 1.43)	1.16 (1.07 to 1.25)	0.32	+	+	
40	AVADO	OS	1.03 (0.70 to 1.33)	0.96 (0.77 to 1.20)	0.35	+	+	

			Effect Estimates (95% CI)			Agreement		
Study No.	Trial name	Outcome	RCT	TTE	SD[d]	SDA	EA	SS
38	EMILIA	OS	0.68 (0.55 to 0.85)	0.64 (0.52 to 0.77)	0.41	+	+	+
19	COST	OS	0.91 (0.68 to 1.21)	0.99 (0.92 to 1.07)	0.55	+	+	P
35	PALOMA-2	OS	0.96 (0.78 to 1.18)	0.81 (0.72 to 0.92)	1.38	+	+	+
36	MONALEESA-2	OS	0.76 (0.63 to 0.93)	0.55 (0.39 to 0.79)	1.57	+		+
3	EORTC	OS	1.02 (0.80 to 1.29)	1.26 (1.20 to 1.33)	1.69	+	+	+
41	BOLERO-2	OS	0.89 (0.73 to 1.10)	1.25 (1.06 to 1.47)	2.54			
37	CLEOPATRA	OS	0.68 (0.56 to 0.84)	0.44 (0.36 to 0.55)	2.91			+
104	KEYNOTE-042	OS	0.73 (0.63 to 0.86)	0.44 (0.34 to 0.57)	3.29			+
102	B-31 and N9831	OS	0.67 (0.48 to 0.93)	0.29 (0.20 to 0.40)	3.43			+
46	KEYNOTE-189	OS	0.49 (0.38 to 0.64)	0.95 (0.78 to 1.16)	3.96			+
5	DOPPS	Mortality	1.04 (0.83 to 1.30)	0.97 (0.94 to 0.99)	0.6	+	+	P
11	VALIDATE	Mortality	1.05 (0.78 to 1.41)	1.21 (0.88 to 1.54)	0.68	+	+	+
92	STEP-HFpEF DM	Mortality	0.40 (0.15 to 0.92)	0.57 (0.47 to 0.69)	0.75	+	+	+
69	DUPL-RECORD1	DVT, PE, Death	0.25 (0.14 to 0.47)	0.17 (0.10 to 0.29)	0.94	+	+	+
73	DUPL-DAPA-CKD	ESRD/Death	0.61 (0.51 to 0.72)	0.80 (0.52 to 1.26)	1.12	+		
22	TASTE	Mortality	0.94 (0.78 to 1.15)	1.09 (0.96 to 1.24)	1.25	+	+	
94	BIG 1-98	Mortality	0.91 (0.75 to 1.11)	1.09 (0.89 to 1.34)	1.25	+	+	
93	SUMMIT	Mortality	0.62 (0.41 to 0.95)	0.39 (0.22 to 0.72)	1.25	+		+
17	MERINO	Mortality	3.69 (1.48 to 10.41)	1.31 (0.40 to 4.26)	1.32	+		
25	COIITSS	Mortality	1.07 (0.88 to 1.30)	0.93 (0.89 to 0.97)	1.38	+	+	P
6	NCT00478205	Mortality	0.84 (0.64 to 1.00)	1.20 (1.07 to 1.33)	2.82			
74	DUPL-PARADIGM-HF	HHF/ Mortality	0.80 (0.73 to 0.87)	1.02 (0.91 to 1.14)	3.33			
**Respiratory**								
79	DUPL-INSPIRE	COPD exacerbation	0.97 (0.84 to 1.12)	0.93 (0.90 to 0.96)	0.56	+	+	+
76	DUPL-D5896	Asthma related death	1.07 (0.70 to 1.65)	1.38 (0.90 to 2.13)	0.82	+	+	+
75	DUPL-PO4334	Asthma exacerbation	0.56 (0.44 to 0.72)	0.78 (0.62 to 0.97)	1.95	+		+

			Effect Estimates (95% CI)			Agreement		
Study No.	Trial name	Outcome	RCT	TTE	SD[d]	SDA	EA	SS
78	DUPL-POET-COPD	COPD exacerbation	0.83 (0.77 to 0.90)	1.02 (0.93 to 1.12)	3.33			
77	DUPL-IMPACT	COPD exacerbation	0.85 (0.80 to 0.90)	1.13 (1.04 to 1.23)	5.44			
**Stroke**								
106	ROCKET AF-Himmelreich	Stroke	0.85 (0.70 to 1.02)	0.90 (0.57 to 1.43)	0.23	+	+	P
63	DUPL-RE-LY	Stroke	0.66 (0.53 to 0.82)	0.73 (0.60 to 0.90)	0.66	+	+	+
64	DUPL-ROCKET AF	Stroke	0.79 (0.66 to 0.96)	0.70 (0.62 to 0.80)	1.05	+	+	+
105	ARISTOTLE-Himmelreich	Stroke	0.79 (0.66 to 0.95)	0.57 (0.34 to 0.94)	1.18	+		+
2	ROCKET AF-Althunian	Stroke	0.88 (0.75 to 1.03)	1.04 (0.84 to 1.30)	1.21	+		
62	DUPL-ARISTOTLE	Stroke	0.79 (0.66 to 0.95)	0.68 (0.61 to 0.76)	1.38	+	+	+
49	ARISTOTLE-Powell	Stroke	0.79 (0.66 to 0.95)	0.98 (0.82 to 1.19)	1.62	+		
7	ACST-2	Stroke	0.35 (0.19 to 0.63)	0.72 (0.53 to 0.99)	2.09			+
8	ACST-1	Stroke	0.47 (0.28 to 0.78)	0.97 (0.67 to 1.42)	2.24			
**Others**								
84	ASSESS	Annualized relapse rate	0.59 (0.37 to 0.95)	0.62 (0.53 to 0.72)	0.2	+	+	+
86	CONFIRM	Annualized relapse rate	0.76 (0.56 to 1.03)	0.79 (0.69 to 0.90)	0.23	+	+	
13	SWEFOT	Good response	1.59 (1.10 to 2.30)	1.48 (0.98 to 2.24)	0.25	+	+	
28	PALOMA-2	Time to Next Treatment	0.64 (0.52 to 0.78)	0.62 (0.56 to 0.68)	0.28	+	+	+
88	REGARD	Annualized relapse rate	1.03 (0.85 to 1.25)	1.00 (0.93 to 1.07)	0.28	+	+	
12	CLEAR-Yiu	PASI >90	1.24 (1.11 to 1.37)	1.28 (1.06 to 1.55)	0.29	+	+	+
10	SONIC2	Treatment failure	0.77 (0.62 to 0.96)	0.73 (0.60 to 0.90)	0.35	+	+	+
89	RIFUND-MS	Annualized relapse rate	0.19 (0.06 to 0.62)	0.25 (0.15 to 0.57)	0.4	+	+	+
51	EUFEST 1	Discontinuation	0.28 (0.18 to 0.43)	0.32 (0.24 to 0.43)	0.5	+	+	+
85	BEYOND	Annualized relapse rate	1.06 (0.89 to 1.26)	1.00 (0.94 to 1.07)	0.62	+	+	
50	TRANSFORMS-Signori	Annualized relapse rate	0.49 (0.37 to 0.64)	0.55 (0.45 to 0.68)	0.66	+	+	+
90	TENERE	Annualized relapse rate	1.20 (0.62 to 2.30)	0.95 (0.83 to 1.07)	0.69	+	+	

Study No.	Trial name	Outcome	Effect Estimates (95% CI)		SD[d]	Agreement		
			RCT	TTE		SDA	EA	SS
1	PreVent	SpO_2<80%	0.48 (0.30 to 0.80)	0.60 (0.40 to 0.90)	0.69	+	+	+
9	SONIC	Treatment failure	0.78 (0.62 to 0.97)	0.71 (0.62 to 0.82)	0.7	+	+	+
82	MiTy	Perinatal events	1.02 (0.83 to 1.26)	0.92 (0.81 to 1.03)	0.84	+	+	
43	HORIZON-D'Andrea	Fracture	0.59 (0.42 to 0.83)	0.72 (0.51 to 0.92)	0.87	+	+	+
52	EUFEST 2	Discontinuation	0.52 (0.35 to 0.76)	0.41 (0.28 to 0.59)	0.87	+	+	+
72	DUPL-HORIZON-PFT	Fracture	0.59 (0.42 to 0.83)	0.72 (0.55 to 0.94)	0.9	+	+	+
21	ORALS	New malignancy	1.48 (1.04 to 2.09)	1.17 (0.85 to 1.62)	0.97	+	+	+
91	TRANSFORMS-Gavoille	Annualized relapse rate	0.48 (0.34 to 0.70)	0.60 (0.53 to 0.69)	1.14	+	+	+
103	OPTIMAP	drug survival	1.27 (0.88 to 1.83)	1.01 (0.92 to 1.12)	1.18	+	+	
14	GRADE	HbA1c>7	0.69 (0.63 to 0.76)	0.55 (0.41 to 0.73)	1.47	+		+
15	GRADE	HbA1c>7	0.79 (0.72 to 0.88)	1.03 (0.94 to 1.13)	3.82			
87	OPERA	Annualized relapse rate	0.53 (0.43 to 0.66)	0.20 (0.14 to 0.29)	4.52			+
16	GRADE	HbA1c>7	1.15 (1.04 to 1.27)	0.57 (0.43 to 0.75)	4.65			

[a] The GRADE study is a multiple-arm RCT that has been split into three pairwise comparisons: (1) Liraglutide vs Sitagliptin; (2) Sitagliptin vs Glimepiride; (3) Liraglutide vs Glimepiride.

[b] The EUFEST study is a multiple-arm RCT that has been split into 2 pairwise comparisons: (1) Olanzapine; (2) Quetiapine.

[c] The RCT-DUPLICATE study compared the effect estimates among 29 RCTs and corresponding emulation with the observational claim database, study No. 53-82.

[d] The standardised difference calculations are available in the Methods section. A standardised difference of 1.00 indicates that the effect estimates from the RCT and the database study are 1 standard deviation apart. Assuming an α level of .05 and assuming that both the database and RCT results are based on large samples, the null hypothesis of no difference would be rejected whenever $|z|>1.96$.

[e] We retained D’Andrea et al. for its trial-specific analyses (two claims databases; 18-month follow-up aligned to real-world adherence)

General Abbreviations: EA, estimate agreement, adjusted database study point estimates falling within the 95% CI of the corresponding randomised clinical trial (RCT) result; SS, statistical significance. Positive represents full agreement for statistical significance, adjusted emulation study and RCT estimates and CIs on the same side of null; MACE major adverse cardiovascular events; P represents partial

agreement for statistical significance, meeting the prespecified noninferiority criteria even though the database study may have indicated superiority; SD, standardised difference, SDA, standardised difference agreement; SS, statistical significant; TTE, target trial emulation; VTE, venous thromboembolism.

Abbreviations for outcomes: 3p MACE, a composite of nonfatal stroke, nonfatal myocardial infarction, and cardiovascular death; AF, atrial fibrillation; CKD, chronic kidney disease; COPD, chronic obstructive pulmonary disease; DOAC, direct oral anticoagulant; DPP4i, dipeptidyl peptidase 4 inhibitors; eGFR, estimated glomerular filtration rate; ESRD, end-stage renal disease; HbA1c (or HA1c), glycated haemoglobin; HHF, hospitalisation for heart failure; MACE, major adverse cardiovascular events, including nonfatal stroke, nonfatal myocardial infarction, and all-cause death; OS, overall survival; PASI, Psoriasis Area and Severity Index; PE, pulmonary embolism; SpO_2, peripheral oxygen saturation; Stroke, cerebrovascular accident (CVA); VTE, venous thromboembolism, including deep vein thrombosis (DVT) and pulmonary embolism (PE); VTE/VTE-related death, venous thromboembolism or death due to VTE; DVT, deep vein thrombosis.

Table C. Comparator, and outcome in TTE-RCT pairs

	RCT			TTE		
**Trial**	**Exposure**	**Comparator**	**Outcome**	**Exposure**	**Comparator**	**Outcome**
PreVent	Bag-mask ventilation	No ventilation	Lowest SpO_2 during intubation; severe hypoxemia (SpO_2 <80%)	Bag-mask or NIV ventilation	No ventilation	Lowest SpO_2 during intubation; severe hypoxemia (SpO_2 <80%)
ROCKET AF-Althunian	Rivaroxaban	Warfarin	Stroke/Systemic Embolism	Rivaroxaban	Warfarin	Stroke/Systemic Embolism
EORTC	Radical nephrectomy + lymph node dissection	Radical nephrectomy alone	OS	Radical nephrectomy + lymph node dissection	Radical nephrectomy alone	OS
IDEA	3 months adjuvant FOLFOX or CAPOX	6 months adjuvant FOLFOX or CAPOX	OS	3–5 months adjuvant FOLFOX or CAPOX	6 months adjuvant FOLFOX or CAPOX	OS
IDEAL	Dialysis initiation at eGFR 10–14	Dialysis initiation at eGFR 5–7	Mortality	Dialysis initiation at eGFR 10–14	Dialysis initiation at eGFR 5–7	Mortality
PIVOTAL	Proactive high-dose IV iron (400mg/month)	Reactive low-dose IV iron (0–400mg/month based on labs)	Mortality	Proactive high-dose IV iron	Reactive low-dose IV iron	Mortality
ACST	Immediate carotid endarterectomy (CEA)	Deferred CEA	stroke	Early CEA (within 1 year)	Initial medical therapy	stroke
ACAS	Carotid endarterectomy (CEA) + medical therapy	Medical therapy	Ipsilateral stroke or perioperative stroke/death	CEA	Medical therapy	Fatal and nonfatal stroke (5-year risk)
SONIC	Infliximab + Azathioprine	Infliximab	Corticosteroid-free clinical remission	Infliximab + Thiopurine	Infliximab	Composite treatment failure

	RCT			TTE		
**Trial**	**Exposure**	**Comparator**	**Outcome**	**Exposure**	**Comparator**	**Outcome**
SONIC2	Infliximab + Azathioprine	Infliximab	Corticosteroid-free clinical remission	Infliximab + Thiopurines	Infliximab	Treatment failure
VALIDATE	Bivalirudin	Heparin	Composite of death, MI, or major bleeding at 180 days	Bivalirudin	Heparin	Death or MI at 180 days (bleeding not included)
CLEAR-Yiu	Secukinumab	Ustekinumab	PASI 90 at 16 weeks	Secukinumab	Ustekinumab	PASI ≤2 at 12 months
SWEFOT	Infliximab + Methotrexate	Sulfasalazine + Hydroxychloroquine + Methotrexate	EULAR good response at 12 months	Infliximab + Methotrexate	Sulfasalazine + Hydroxychloroquine + Methotrexate	EULAR good response at 9 months
GRADE	Liraglutide + Metformin	Glimepiride + Metformin	HbA1c	Liraglutide + Metformin	Glimepiride + Metformin	HbA1c
GRADE	Sitagliptin + Metformin	Glimepiride + Metformin	HbA1c	Sitagliptin + Metformin	Glimepiride + Metformin	HbA1c
GRADE	Liraglutide + Metformin	Sitagliptin + Metformin	HbA1c	Liraglutide + Metformin	Sitagliptin + Metformin	HbA1c
MERINO	Piperacillin-Tazobactam	Meropenem	30-day all-cause mortality	Piperacillin-Tazobactam	Meropenem	25-day all-cause mortality
CHAARTED	Docetaxel + ADT	ADT alone	OS	Docetaxel + ADT	ADT alone	OS
COST	Laparoscopy-assisted colectomy (LAC)	Open colectomy (OC)	OS	LAC	OC	OS
EMPA-REG-Jang	Empagliflozin	Placebo	3p MACE	Empagliflozin	DPP4i	3p MACE
ORALS	Tofacitinib	TNF Inhibitor	Cancer; MACE	Tofacitinib	TNF Inhibitor	Cancer
TASTE	PCI + thrombus aspiration	PCI alone	All-cause death and MI	PCI + thrombus aspiration	PCI alone	All-cause death and MI
PARSIFAL	Palbociclib + Fulvestrant	Palbociclib + Letrozole	OS	Palbociclib + Fulvestrant	Palbociclib + Letrozole	OS

	RCT			TTE		
**Trial**	**Exposure**	**Comparator**	**Outcome**	**Exposure**	**Comparator**	**Outcome**
E2100	Paclitaxel + Bevacizumab	Paclitaxel alone	OS	Paclitaxel + Bevacizumab	Paclitaxel alone	OS
COIITSS	Hydrocortisone + Fludrocortisone	Hydrocortisone alone	Mortality	Hydrocortisone + Fludrocortisone	Hydrocortisone alone	Mortality or discharge to hospice
ACCORD	ARB-based antihypertensive regimen	ACEI-based antihypertensive regimen	3p MACE	ARB	ACEI	3p MACE
SPRINT	ARB-based antihypertensive regimen	ACEI-based antihypertensive regimen	3p MACE	ARB	ACEI	3p MACE
PALOMA-2	Palbociclib + Letrozole	Placebo + Letrozole	PFS	Palbociclib + Letrozole	Letrozole only	Time to Next Treatment (TTNT)
AMPLIFY	Apixaban	Enoxaparin/ warfarin	VTE / VTE Related Death	Apixaban	Heparin + Warfarin	VTE / VTE Related Death
RE-COVER II	Dabigatran	Heparin + Warfarin	VTE / VTE Related Death	Dabigatran	Heparin + Warfarin	VTE / VTE Related Death
Hokusai-VTE	Edoxaban (after heparin)	Heparin + Warfarin	VTE / VTE Related Death	Edoxaban	Heparin + Warfarin	VTE / VTE Related Death
EINSTEIN-DVT	Rivaroxaban	Enoxaparin/ VKA	VTE	Rivaroxaban	Warfarin	VTE
EINSTEIN-PE	Rivaroxaban	Warfarin	VTE	Rivaroxaban	Warfarin	VTE
PALOMA-3	Palbociclib + Fulvestrant	Placebo + Fulvestrant	PFS, OS	Palbociclib + Fulvestrant	Fulvestrant	OS
PALOMA-2	Palbociclib + Letrozole	Placebo + Letrozole	PFS, OS	Palbociclib + Letrozole	Letrozole	OS
MONALEESA-2	Ribociclib + Letrozole	Placebo + Letrozole	PFS, OS	Ribociclib + Letrozole	Letrozole	OS
CLEOPATRA	Pertuzumab + Trastuzumab + Docetaxel	Placebo + Trastuzumab + Docetaxel	PFS, OS	Pertuzumab + Trastuzumab + Docetaxel	Trastuzumab + Docetaxel	OS

	RCT			TTE		
Trial	Exposure	Comparator	Outcome	Exposure	Comparator	Outcome
EMILIA	T-DM1	Lapatinib + Capecitabine	PFS, OS	T-DM1	Lapatinib + Capecitabine	OS
RIBBON-1	Bevacizumab + Chemotherapy	Placebo + Chemotherapy	PFS, OS	Bevacizumab + Chemotherapy	Chemotherapy	OS
AVADO	Bevacizumab + Docetaxel	Placebo + Docetaxel	PFS, OS	Bevacizumab + Docetaxel	Docetaxel	OS
BOLERO-2	Everolimus + Exemestane	Placebo + Exemestane	PFS, OS	Everolimus + Exemestane	Exemestane	OS
ONTARGET-Baptiste	Telmisartan	Ramipril	3p MACE	ARB (any)	ACEi (any)	3p MACE
HORIZON-D'Andrea	Zoledronic Acid	Placebo	Hip Fracture	Zoledronic Acid	Raloxifene	Hip Fracture
ISAR-REACT5	Prasugrel	Ticagrelor	3p MACE	Prasugrel	Ticagrelor	3p MACE
REDUCE-AMI	Beta-blockers (Metoprolol or Bisoprolol)	No beta-blocker	3p MACE	Beta-blocker prescription (within 30d)	No beta-blocker prescription	3p MACE
KEYNOTE-189	Pembrolizumab + Chemo (PEM+PLAT)	Placebo + Chemo (PEM+PLAT)	OS	Pembrolizumab + Chemo	Chemo only	OS
INVESTED	High-dose trivalent IIV	Standard-dose quadrivalent IIV	3p MACE	High-dose trivalent IIV	Standard-dose quadrivalent IIV	3p MACE
MINT	Liberal strategy (Hb <10 g/dL)	Restrictive strategy	3p MACE	Liberal strategy (Hb <10 g/dL)	Restrictive strategy	3p MACE
ARISTOTLE-Powell	Apixaban	Warfarin	Stroke/Systemic Embolism	Apixaban	Warfarin	Stroke/Systemic Embolism
TRANSFORMS-Signori	Fingolimod	Interferon	Annualized relapse rate	Fingolimod	Interferon	Annualized relapse rate
EUFEST 1	Olanzapine	Haloperidol	discontinuation	Olanzapine	First-generationagents: haloperidol, fluphenazine, perphenazine	discontinuation

Trial	RCT			TTE		
	**Exposure**	**Comparator**	**Outcome**	**Exposure**	**Comparator**	**Outcome**
EUFEST 2	Quetiapine	Haloperidol	discontinuation	Quetiapine	First-generationagents: haloperidol, fluphenazine, perphenazine	discontinuation
DUPL-LEADER	Liraglutide	Placebo	3p MACE	Liraglutide	DPP4i	3p MACE
DUPL-DECLARE-TIMI58	Dapagliflozin	Placebo	HHF + cardiovascular death	Dapagliflozin	DPP4i	HHF + cardiovascular death
DUPL-EMPA-REG	Empagliflozin	Placebo	3p MACE	Empagliflozin	DPP4i	3p MACE
DUPL-CANVAS	Canagliflozin	Placebo	3p MACE	Canagliflozin	DPP4i	3p MACE
DUPL-CARMELINA	Linagliptin	Placebo	3p MACE	Linagliptin	Sulfonylureas	3p MACE
DUPL-TECOS	Sitagliptin	Placebo	3p MACE+ angina	Sitagliptin	Sulfonylureas	3p MACE+ angina
DUPL-SAVOR-TIMI	Saxagliptin	Placebo	3p MACE	Saxagliptin	Sulfonylureas	3p MACE
DUPL-TRITON-TIMI	Prasugrel	Clopidogrel	3p MACE	Prasugrel	Clopidogrel	3p MACE
DUPL-PLATO	Ticagrelor	Clopidogrel	3p MACE	Ticagrelor	Clopidogrel	3p MACE
DUPL-ARISTOTLE	Apixaban	Warfarin	Stroke/Systemic Embolism	Apixaban	Warfarin	Stroke/Systemic Embolism
DUPL-RE-LY	Dabigatran	Warfarin	Stroke/Systemic Embolism	Dabigatran	Warfarin	Stroke/Systemic Embolism
DUPL-ROCKET AF	Rivaroxaban	Warfarin	Stroke/Systemic Embolism	Rivaroxaban	Warfarin	Stroke/Systemic Embolism
DUPL-EINSTEIN DVT	Rivaroxaban	Enoxaparin/ VKA	VTE	Rivaroxaban	Warfarin	VTE

Trial	RCT			TTE		
	Exposure	Comparator	Outcome	Exposure	Comparator	Outcome
DUPL-EINSTEIN PE	Rivaroxaban	Warfarin	VTE	Rivaroxaban	Warfarin	VTE
DUPL-RE-COVER II	Dabigatran	Warfarin	VTE / VTE Related Death	Dabigatran	Warfarin	VTE / VTE Related Death
DUPL-AMPLIFY	Apixaban	Enoxaparin/ warfarin	VTE / VTE Related Death	Apixaban	Warfarin	VTE / VTE Related Death
DUPL-RECORD1	Rivaroxaban	Enoxaparin	DVT, PE, Death	Rivaroxaban	Enoxaparin	DVT, PE, Death
DUPL-TRANSCEND	Telmisartan	Placebo	3p MACE + HHF	Telmisartan+Loop/CCB/TZ	Loop/CCB/ TZ	3p MACE + HHF
DUPL-ONTARGET	Telmisartan	Ramipril	3p MACE + HHF	Telmisartan	Ramipril	3p MACE + HHF
DUPL-HORIZON-PFT	Zoledronic Acid	Placebo	Hip Fracture	Zoledronic Acid	Raloxifene	Hip Fracture
DUPL-DAPA-CKD	Dapagliflozin (SGLT2)	Placebo	Sustained decline in eGFR/ESRD/Death	Dapagliflozin	Sitagliptin	ESRD/Death
DUPL-PARADIGM-HF	Sacubitril/ Valsartan	Enalapril	HHF/ Cardiovascular death	Sacubitril/ Valsartan	ACEi	HHF/ Mortality
DUPL-PO4334	Mometasone furoate/formoterol	Formoterol	Asthma exacerbation	Mometasone furoate/formoterol	Formoterol	Asthma exacerbation
DUPL-D5896	Budesonide + formoterol	Budenoside	Asthma related death, intubation, hosp	Budesonide + formoterol	Budenoside	Asthma related death, intubation, hosp
DUPL-IMPACT	Fluticasone, umeclidinium, vilanterol	Fluticasone, vilanterol	Moderate or severe COPD exacerbation	Fluticasone, umeclidinium, vilanterol	Fluticasone, vilanterol	Moderate or severe COPD exacerbation
DUPL-POET-COPD	Tiotropium	Salmeterol	Moderate or severe COPD exacerbation	Tiotropium	Salmeterol	Moderate or severe COPD exacerbation

Trial	RCT			TTE		
	**Exposure**	**Comparator**	**Outcome**	**Exposure**	**Comparator**	**Outcome**
DUPL-INSPIRE	Advair	Tiotropium	Moderate or severe COPD exacerbation	Advair	Tiotropium	Moderate or severe COPD exacerbation
DUPL-CAROLINA	Linagliptin	Glimepiride	3p MACE	Linagliptin	Glimepiride	3p MACE
DUPL-PRONOUNCE	Degarelix	Leuprolide	MACE	Degarelix	Leuprolide	MACE
MiTy	Insulin + Metformin	Insulin + Placebo	Composite of perinatal events	Insulin + metformin	Insulin	Composite of perinatal events
TRANSFORMS-Gavoille	Empagliflozin	Placebo	3p MACE	Empagliflozin	DPP4i	3p MACE
ASSESS	Fingolimod	Glatiramer acetate	ARR	Fingolimod	Glatiramer acetate	ARR
BEYOND	Interferon-β	Glatiramer acetate	ARR	Interferon-β	Glatiramer acetate	ARR
CONFIRM	Dimethyl fumarate (DMF)	Glatiramer acetate	ARR	DMF	Glatiramer acetate	ARR
OPERA	Ocrelizumab	Interferon-β	ARR	Ocrelizumab	Interferon-β	ARR
REGARD	Interferon-β	Glatiramer acetate	ARR	Interferon-β	Glatiramer acetate	ARR
RIFUND-MS	Rituximab	DMF	ARR	Rituximab	DMF	ARR
TENERE	Teriflunomide	Interferon-β	ARR	Teriflunomide	Interferon-β	ARR
TRANSFORMS-Gavoille	Fingolimod	Interferon-β	ARR	Fingolimod	Interferon-β	ARR
STEP-HFpEF DM	Semaglutide	Placebo	Composite of HF hospitalization	Semaglutide	Sitagliptin	Composite of HF hospitalization
SUMMIT	Tirzepatide	Placebo	Composite of HF hospitalization	Tirzepatide	Sitagliptin	Composite of HF hospitalization
BIG 1-98	Tirzepatide	Placebo	Composite of HF hospitalization	Tirzepatide	Sitagliptin	Composite of HF hospitalization
CAROLINA-Ko	Linagliptin	Glimepiride	3-point MACE	Linagliptin	Glimepiride	3-point MACE
CARMELINA-Ko	Linagliptin	Placebo	3-point MACE	Linagliptin	Glimepiride	3-point MACE

Trial	RCT			TTE		
	Exposure	Comparator	Outcome	Exposure	Comparator	Outcome
TECOS-Ko	Sitagliptin	Placebo	4P-MACE	Sitagliptin	sulfonylureas	4P-MACE
SAVOR-TIMI 53-Ko	Saxagliptin	Placebo	3-point MACE	Saxagliptin	sulfonylureas	3-point MACE
DECLARE-TIMI 58-Ko	Dapagliflozin	Placebo	3-point MACE	Dapagliflozin	DPP-4 inhibitor	3-point MACE
EMPA-REG-Ko	Empagliflozin	Placebo	3-point MACE	Empagliflozin	DPP-4 inhibitor	3-point MACE
PRASFIT-ACS	prasugrel + aspirin	Clopidogrel + aspirin	MACE	prasugrel + aspirin	Clopidogrel + aspirin	MACE
B-31 and N9831	trastuzumab + chemotherapy	Chemotherapy alone	DFS, OS	trastuzumab + chemotherapy	Chemotherapy alone	DFS, OS
OPTIMAP	Adalimumab+ methotrexate	Adalimumab	Drug survival	Adalimumab+ methotrexate	Adalimumab	Drug survival
KEYNOTE-042	Pembrolizumab	Platinum-based chemotherapy	OS, PFS	Pembrolizumab	Platinum-based chemotherapy	OS; PFS
ARISTOTLE-Himmelreich	Apixaban	Warfarin	Stroke	Apixaban	Warfarin	Stroke
ROCKET AF-Himmelreich	Rivaroxaban	Warfarin	Stroke	Rivaroxaban	Warfarin	Stroke

Abbreviations: 3p MACE, composite of nonfatal stroke, nonfatal myocardial infarction, and cardiovascular death; AF, atrial fibrillation; CKD, chronic kidney disease; COPD, chronic obstructive pulmonary disease; DOAC, direct oral anticoagulant; DPP4i, dipeptidyl peptidase 4 inhibitors; eGFR, estimated glomerular filtration rate; ESRD, end-stage renal disease; HbA1c (or HA1c), glycated haemoglobin; HHF, hospitalization for heart failure; MACE, major adverse cardiovascular events, including nonfatal stroke, nonfatal myocardial infarction, and all-cause death; OS, overall survival; PASI, Psoriasis Area and Severity Index; PE, pulmonary embolism; SpO_2, peripheral oxygen saturation; Stroke, cerebrovascular accident (CVA); VTE, venous thromboembolism, including deep vein thrombosis (DVT) and pulmonary embolism (PE); VTE/VTE-related death, venous thromboembolism or death due to VTE; DVT, deep vein thrombosis.

Table D. Multivariable regression coefficients (95% confidence intervals) for the association between candidate characteristics and differences in log ratios across 106 TTE-RCT pairs

**Multivariable model** #	**Univariate Coefficient*** **(95% CI)**	**P value**
Delayed effect (no)	-0.218 (-0.381 to -0.055)	0.009
Difference in percentage of sex	0.004 (-0.009 to 0.001)	0.115
Treatment started in hospital (no)	-0.101 (-0.228 to 0.025)	0.152

#The multivariable model was constructed using stepwise linear regression, selecting from nine candidate variables after excluding 'Close emulation' and 'Placebo control' due to multicollinearity. Three variables were retained in the final model. The model explained approximately 10% of the variance in log ratio differences (marginal $R^2 = 0.104$), and study-level clustering accounted for minimal variation (ICC = 0.091).

eAppendix Figures

Figure A. Summary of bias of the TTE with ROBIN-I tool
Abbreviations: TTE, target trial emulation; RoB 2, Cochrane Risk of Bias Tool version 2.0.

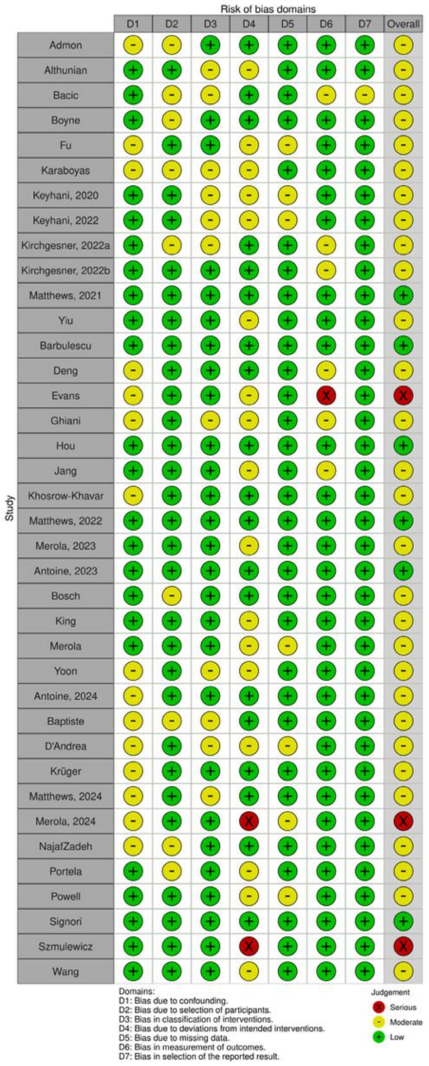

Figure B. Summary of bias of the TTE with ROBIN-I tool
Abbreviations: TTE, target trial emulation; RoB 2, Cochrane Risk of Bias Tool version 2.0.

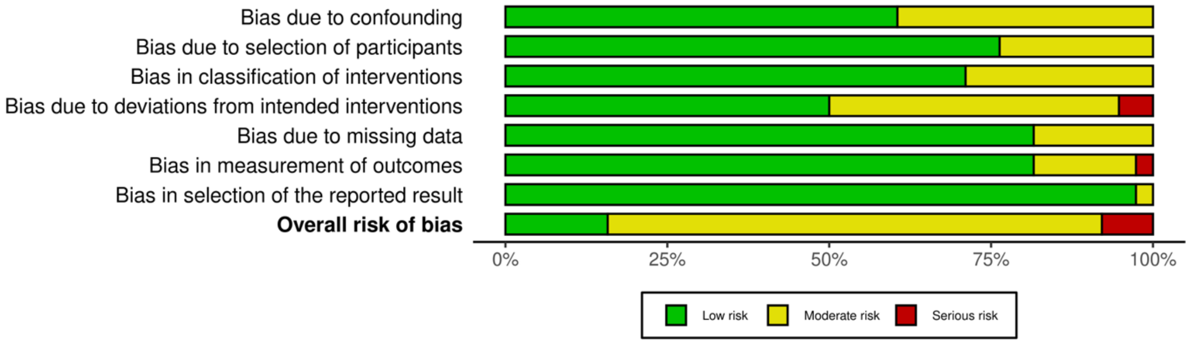

Figure C. Summary of bias of RCTs with RoB 2 tool

Abbreviations: RCT, randomized controlled trials; ROBINS-I, Cochrane Risk Of Bias In Non-randomized Studies of Interventions.

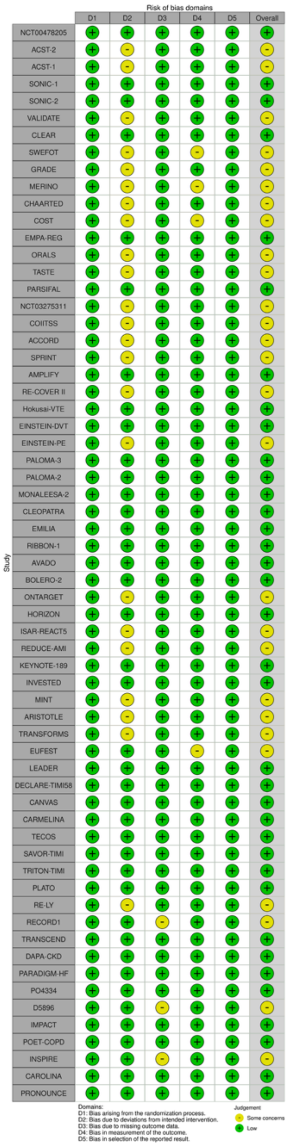

Figure D. Summary of bias of the RCT with RoB 2 tool

Abbreviations: RCT, randomized controlled trials; ROBINS-I, Cochrane Risk Of Bias In Non-randomized Studies of Interventions.

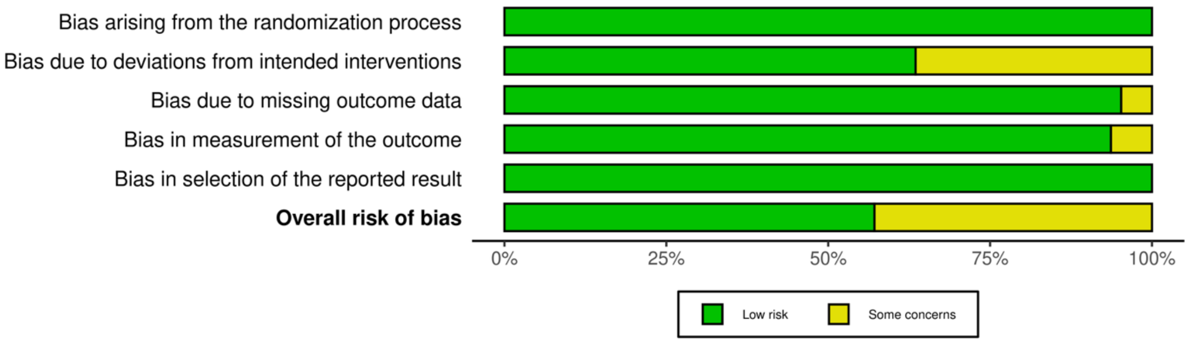

Figure E. Forest plots of ratio of ratios categorised by intervention

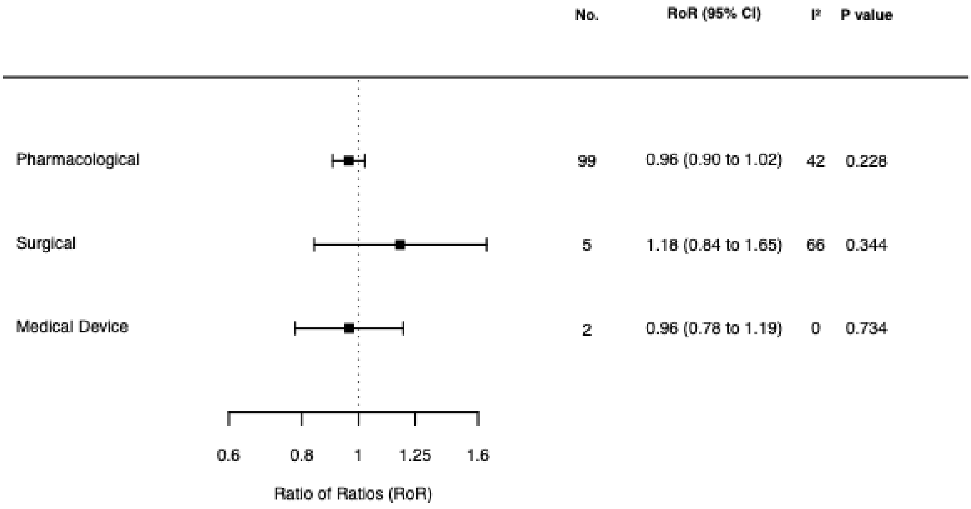

Figure F. Forest plots of ratio of ratios categorised by database region

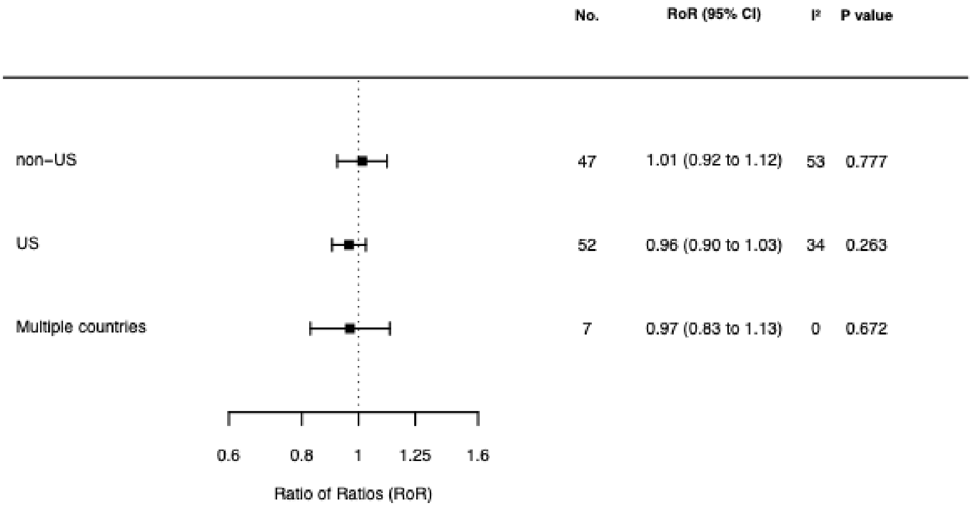

Figure G. Forest plots of ratio of ratios categorised by confounding adjustment methods

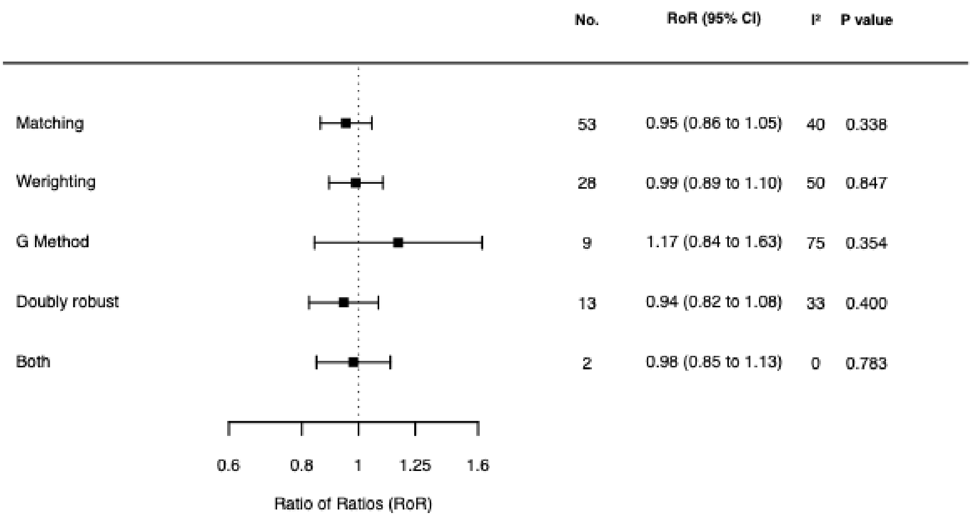

Figure H. Forest plots of ratio of ratios categorised by report metrics

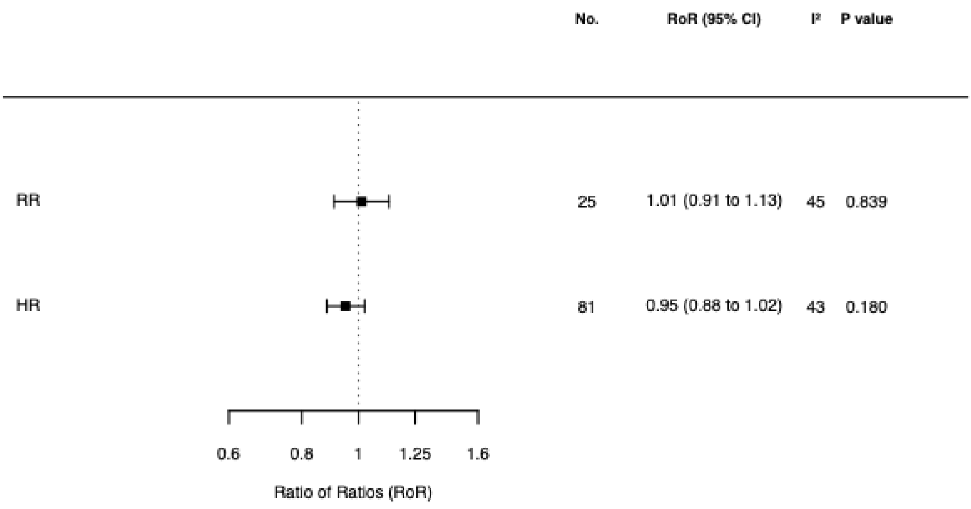

Figure I. Forest plots of ratio of ratios categorised by estimand

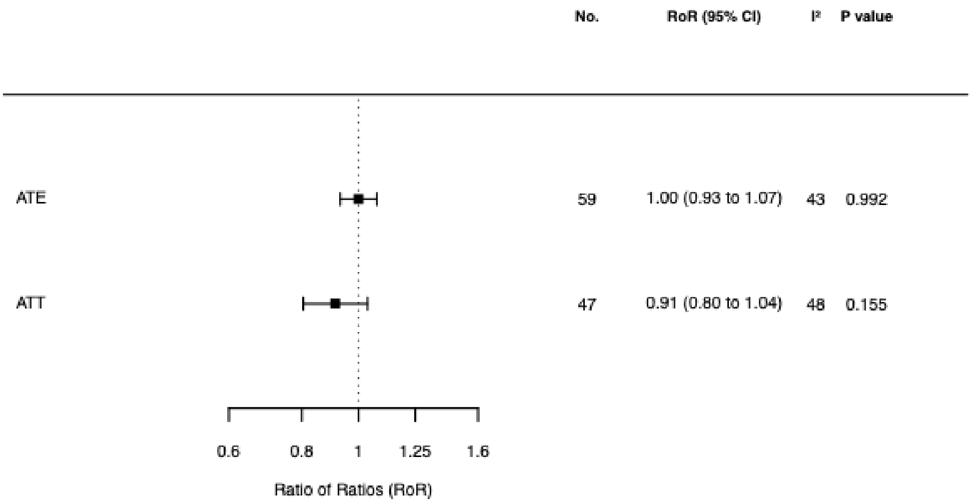

Figure J. Forest plots of ratio of ratios categorised by guideline report

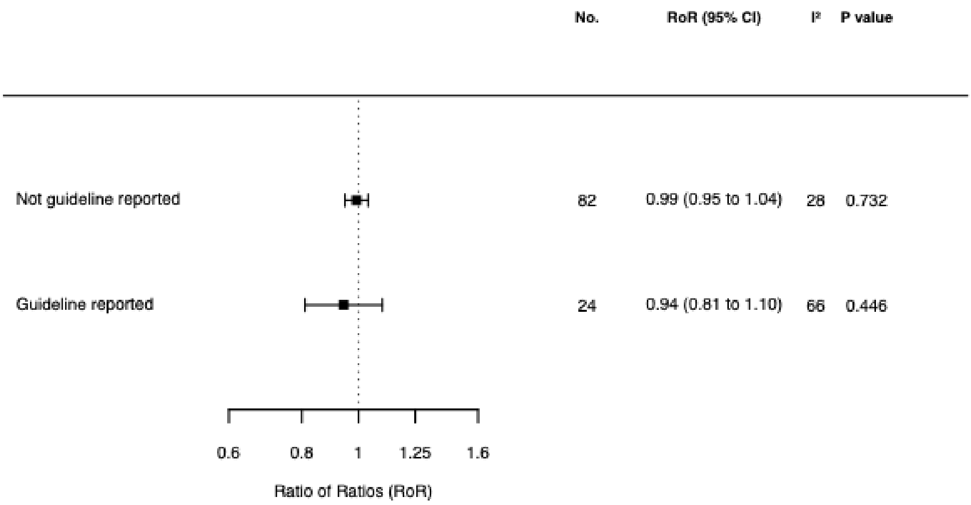

Figure K. Forest plots of ratio of ratios categorised by protocol registration

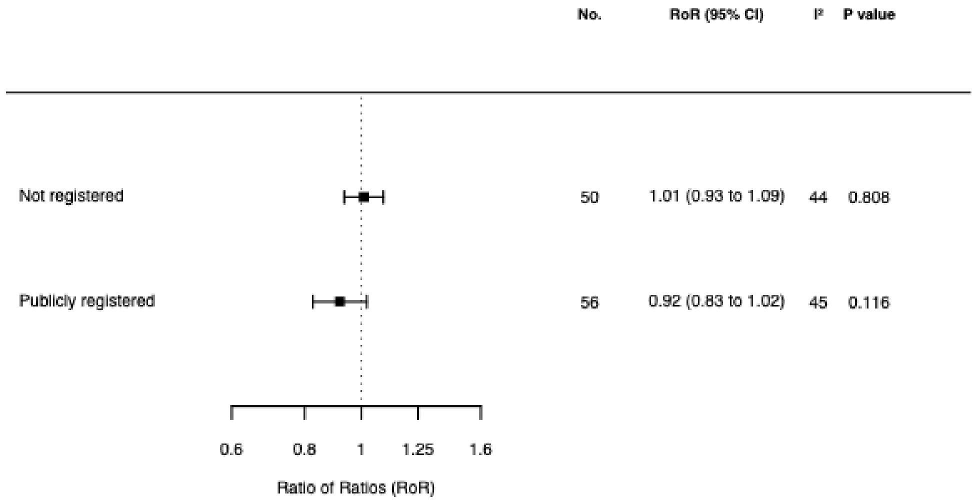

Figure L. Forest plots of ratio of ratios categorised by non-inferior vs superiority

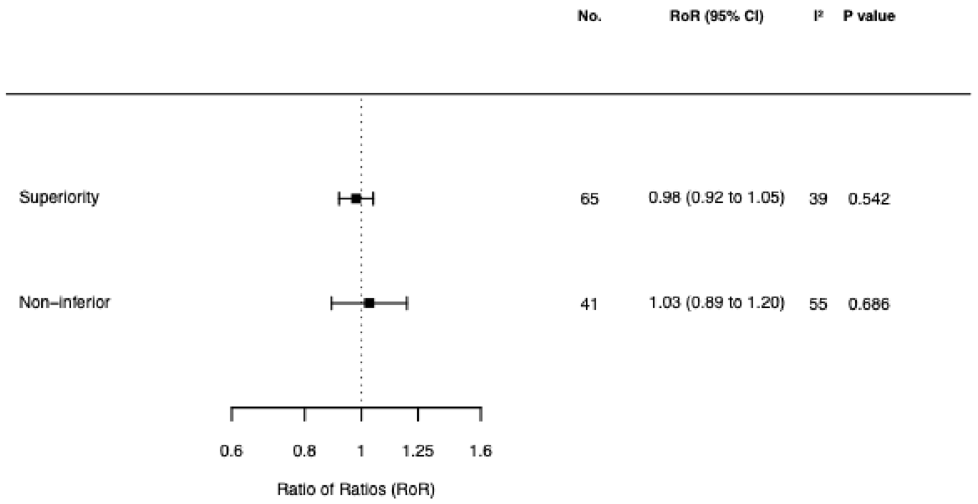

Figure M. Forest plots of ratio of ratios categorised by emulation design

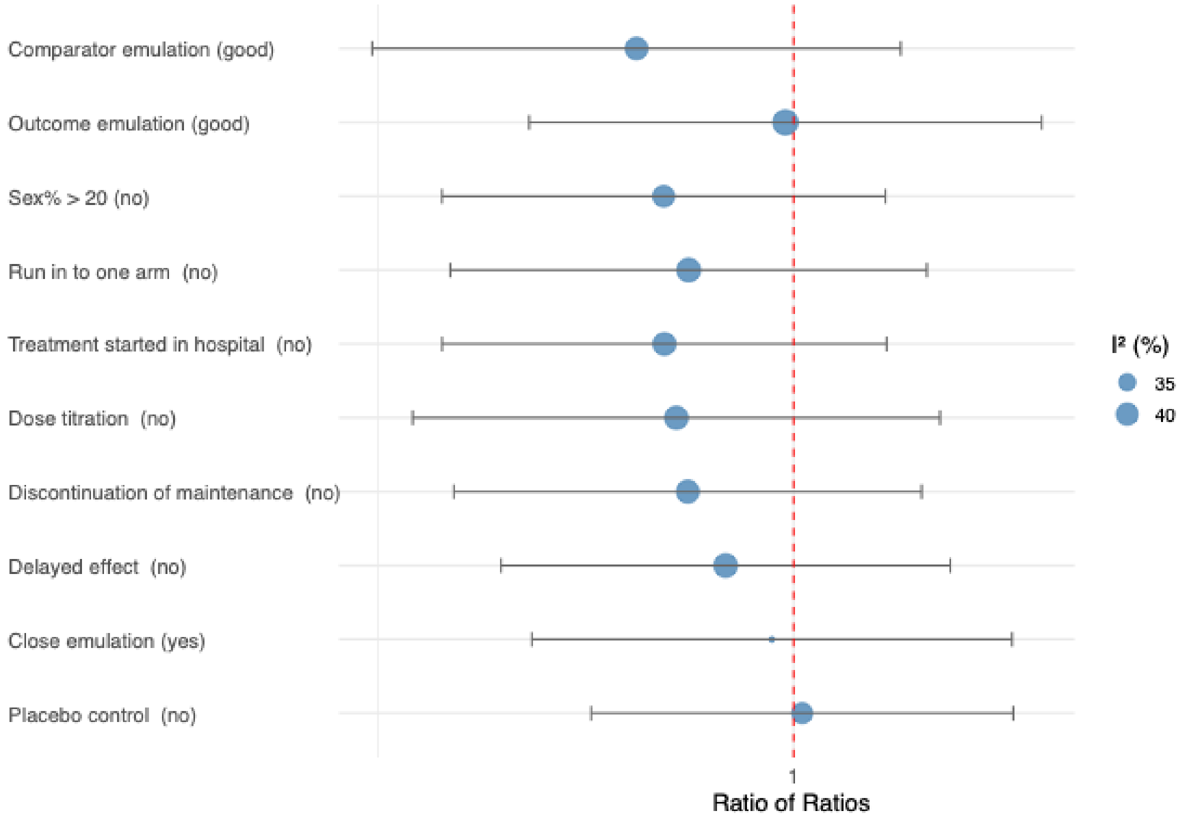

Figure N. (A) Sensitivity analysis of leave-one-out cross validation for 106 pairs. (B) Sensitivity analysis of leave-one-out cross validation for 49 studies.

Note: This plot presents the results of a leave-one-out cross-validation (LOO-CV) sensitivity analysis, assessing the robustness of the overall effect. The study labelled "MERINO" demonstrates a relatively high impact on the results.

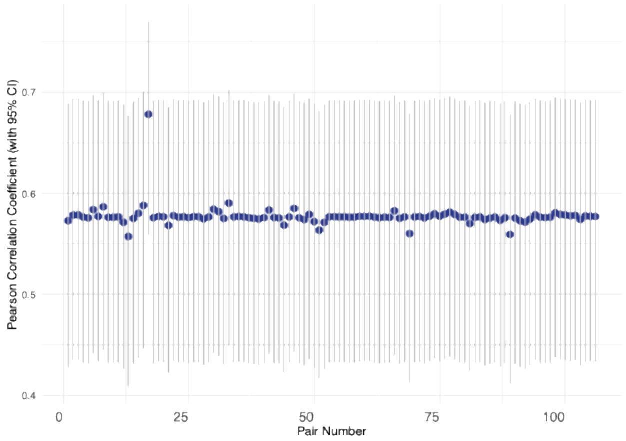

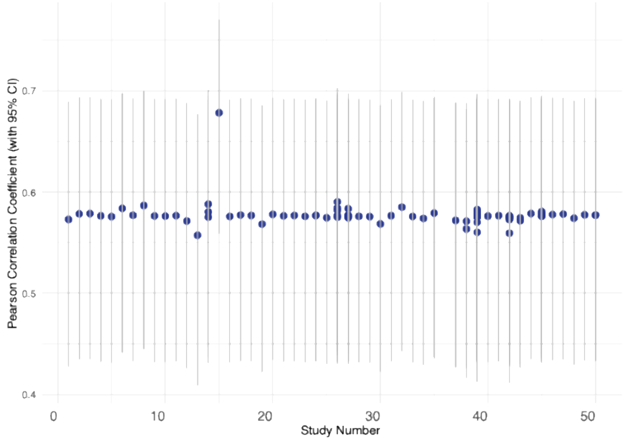

Reference:

1. Admon, A.J., et al., *Emulating a Novel Clinical Trial Using Existing Observational Data. Predicting Results of the PreVent Study.* Ann Am Thorac Soc, 2019. **16**(8): p. 998-1007.
2. Althunian, T.A., et al., *Rivaroxaban was found to be noninferior to warfarin in routine clinical care: A retrospective noninferiority cohort replication study.* Pharmacoepidemiol Drug Saf, 2020. **29**(10): p. 1263-1272.
3. Bacic, J. and B. Gershman, *Emulating Target Clinical Trials of Radical Nephrectomy With or Without Lymph Node Dissection for Renal Cell Carcinoma REPLY.* Urology, 2020. **140**: p. 106-106.
4. Boyne, D.J., et al., *Association of a Shortened Duration of Adjuvant Chemotherapy With Overall Survival Among Individuals With Stage III Colon Cancer.* JAMA Netw Open, 2021. **4**(3): p. e213587.
5. Fu, E.L., et al., *Timing of dialysis initiation to reduce mortality and cardiovascular events in advanced chronic kidney disease: nationwide cohort study.* Bmj, 2021. **375**: p. e066306.
6. Karaboyas, A., et al., *Replicating Randomized Trial Results with Observational Data Using the Parametric g-Formula: An Application to Intravenous Iron Treatment in Hemodialysis Patients.* Clin Epidemiol, 2020. **12**: p. 1249-1260.
7. Keyhani, S., et al., *Comparative Effectiveness of Carotid Endarterectomy vs Initial Medical Therapy in Patients With Asymptomatic Carotid Stenosis.* JAMA Neurol, 2020. **77**(9): p. 1110-1121.
8. Keyhani, S., et al., *Comparative Effectiveness of Carotid Stenting to Medical Therapy Among Patients With Asymptomatic Carotid Stenosis.* Stroke, 2022. **53**(4): p. 1157-1166.
9. Kirchgesner, J., et al., *Calibrating Real-World Evidence Studies Against Randomized Trials: Treatment Effectiveness of Infliximab in Crohn's Disease.* Clin Pharmacol Ther, 2022. **111**(1): p. 179-186.
10. Kirchgesner, J., et al., *Emulation of a randomized controlled trial in ulcerative colitis with US and French claims data: Infliximab with thiopurines compared to infliximab monotherapy.* Pharmacoepidemiology and Drug Safety, 2022. **31**(2): p. 167-175.
11. Matthews, A.A., et al., *Comparing Effect Estimates in Randomized Trials and Observational Studies From the Same Population: An Application to Percutaneous Coronary Intervention.* J Am Heart Assoc, 2021. **10**(11): p. e020357.
12. Yiu, Z.Z.N., et al., *Randomized Trial Replication Using Observational Data for Comparative Effectiveness of Secukinumab and Ustekinumab in Psoriasis: A Study From the British Association of Dermatologists Biologics and Immunomodulators Register.* JAMA Dermatol, 2021. **157**(1): p. 66-73.
13. Barbulescu, A., et al., *Combined Conventional Synthetic Disease Modifying Therapy vs. Infliximab for Rheumatoid Arthritis: Emulating a Randomized Trial in Observational Data.* Clinical Pharmacology & Therapeutics, 2022. **112**(4): p. 836-845.
14. Deng, Y., et al., *Emulating the GRADE trial using real world data: retrospective comparative effectiveness study.* Bmj, 2022. **379**: p. e070717.
15. Evans, R.N., et al., *Emulating the MERINO randomised control trial using data from an observational cohort and trial of rapid diagnostic (BSI-FOO).* Plos One, 2022. **17**(5): p. 12.

16. Ghiani, M., et al., *Bridging the gap between oncology clinical trials and real-world data: evidence on replicability of efficacy results using German claims data.* J Comp Eff Res, 2022. **11**(7): p. 513-521.
17. Hou, J., et al., *Temporal Trends in Clinical Evidence of 5-Year Survival Within Electronic Health Records Among Patients With Early-Stage Colon Cancer Managed With Laparoscopy-Assisted Colectomy vs Open Colectomy.* JAMA Netw Open, 2022. **5**(6): p. e2218371.
18. Jang, H., I.W. Kim, and J.M. Oh, *Using real-world data for supporting regulatory decision making: Comparison of cardiovascular and safety outcomes of an empagliflozin randomized clinical trial versus real-world data.* Frontiers in Pharmacology, 2022. **13**: p. 13.
19. Khosrow-Khavar, F., et al., *Tofacitinib and Risk of Malignancy: Results From the Safety of Tofacitinib in Routine Care Patients With Rheumatoid Arthritis (STAR-RA) Study.* Arthritis Rheumatol, 2022. **74**(10): p. 1648-1659.
20. Matthews, A.A., et al., *Benchmarking Observational Analyses Before Using Them to Address Questions Trials Do Not Answer: An Application to Coronary Thrombus Aspiration.* Am J Epidemiol, 2022. **191**(9): p. 1652-1665.
21. Merola, D., et al., *Oncology Drug Effectiveness from Electronic Health Record Data Calibrated Against RCT Evidence: The PARSIFAL Trial Emulation.* Clin Epidemiol, 2022. **14**: p. 1135-1144.
22. Antoine, A., et al., *Target trial emulation to assess real-world efficacy in the Epidemiological Strategy and Medical Economics metastatic breast cancer cohort.* Jnci-Journal of the National Cancer Institute, 2023. **115**(8): p. 971-980.
23. Bosch, N.A., et al., *Comparative Effectiveness of Fludrocortisone and Hydrocortisone vs Hydrocortisone Alone Among Patients With Septic Shock.* JAMA Intern Med, 2023. **183**(5): p. 451-459.
24. King, J.B., et al., *New Users of Angiotensin II Receptor Blocker-Versus Angiotensin-Converting Enzyme Inhibitor-Based Antihypertensive Medication Regimens and Cardiovascular Disease Events: A Secondary Analysis of ACCORD-BP and SPRINT.* J Am Heart Assoc, 2023. **12**(17): p. e030311.
25. Merola, D., et al., *Effectiveness research in oncology with electronic health record data: A retrospective cohort study emulating the PALOMA-2 trial.* Pharmacoepidemiology and Drug Safety, 2023. **32**(4): p. 426-434.
26. Yoon, D., et al., *Real-world data emulating randomized controlled trials of non-vitamin K antagonist oral anticoagulants in patients with venous thromboembolism.* Bmc Medicine, 2023. **21**(1): p. 13.
27. Antoine, A., et al., *Assessing the real-world effectiveness of 8 major metastatic breast cancer drugs using target trial emulation.* Eur J Cancer, 2024. **213**: p. 115072.
28. Baptiste, P.J., et al., *Cardiorenal effects of angiotensin-converting enzyme inhibitors and angiotensin receptor blockers among people underrepresented in trials: analysis of routinely collected data with emulation of a reference trial (ONTARGET).* American Journal of Epidemiology, 2024: p. 11.
29. D'Andrea, E., et al., *Efficacy versus effectiveness: The HORIZON Pivotal Fracture Trial and its emulation in Claims Data.* Arthritis Rheumatol, 2024.
30. Krüger, N., et al., *Ticagrelor vs Prasugrel for Acute Coronary Syndrome in Routine Care.* JAMA Netw Open, 2024. **7**(12): p. e2448389.
31. Matthews, A.A., et al., *Prospective benchmarking of an observational analysis in the SWEDEHEART registry against the REDUCE-AMI randomized trial.* Eur J Epidemiol, 2024. **39**(4): p. 349-361.

32. Merola, D., et al., *Calibrating Observational Health Record Data Against a Randomized Trial.* JAMA Netw Open, 2024. **7**(9): p. e2436535.
33. Najafzadeh, M., et al., *High-Dose vs. Standard-Dose Influenza Vaccine and Cardiopulmonary Hospitalization or Mortality: Emulating the INVESTED Trial Using Insurance Claims Data.* Clinical Pharmacology & Therapeutics, 2024. **115**(1): p. 126-134.
34. Portela, G.T., et al., *Effect of Four Hemoglobin Transfusion Threshold Strategies in Patients With Acute Myocardial Infarction and Anemia : A Target Trial Emulation Using MINT Trial Data.* Ann Intern Med, 2024. **177**(11): p. 1489-1498.
35. Powell, E.M., et al., *Comparison of oral anticoagulants for stroke prevention in atrial fibrillation using the UK clinical practice research Datalink Aurum: A reference trial (ARISTOTLE) emulation study.* PLoS Med, 2024. **21**(8): p. e1004377.
36. Signori, A., et al., *Emulating randomised clinical trials in relapsing-remitting multiple sclerosis with non-randomised real-world evidence: an application using data from the MSBase Registry.* Journal of Neurology Neurosurgery and Psychiatry, 2024. **95**(7): p. 620-625.
37. Szmulewicz, A.G., et al., *Antipsychotic drugs in first-episode psychosis: a target trial emulation in the FEP-CAUSAL Collaboration.* American Journal of Epidemiology, 2024. **193**(8): p. 1081-1087.
38. Wang, S.V., S. Schneeweiss, and J.M. Franklin, *Emulation of randomized clinical trials with nonrandomized database analyses: results of 32 clinical trials (vol 329, pg 1376, 2023).* Jama-Journal of the American Medical Association, 2024. **331**(14): p. 1236-1236.
39. Yland, J.J., et al., *Perinatal Outcomes Associated With Metformin Use During Pregnancy in Women With Pregestational Type 2 Diabetes Mellitus.* Diabetes Care, 2024. **47**(9): p. 1688-1695.
40. Yazdanfard, P.D.W., et al., *Type 2 diabetes, sodium-glucose cotransporter-2 inhibitors and cardiovascular outcomes: real world evidence versus a randomised clinical trial.* Cardiovasc Diabetol, 2025. **24**(1): p. 371.
41. Gavoille, A., et al., *Target trial emulation to replicate randomised clinical trials using registry data in multiple sclerosis.* J Neurol Neurosurg Psychiatry, 2025.
42. Kruger, N., et al., *Semaglutide and Tirzepatide in Patients With Heart Failure With Preserved Ejection Fraction.* JAMA, 2025. **334**(14): p. 1255-1266.
43. Al-Kassab-Cordova, A., et al., *Endocrine therapies and mortality risk in postmenopausal women with breast cancer: benchmarking an observational analysis against a randomized trial.* Am J Epidemiol, 2025.
44. Ko, H.Y., et al., *Emulating Six Cardiovascular Outcome Trials of Antidiabetic Drugs in Individuals with Type 2 Diabetes: Analyses Based on Real-World Databases from Korea and Taiwan.* Clin Pharmacol Ther, 2025.
45. Huang, L.W., et al., *Effectiveness and Safety of Reduced-Dose Prasugrel in an East Asian Population: PRASFIT-ACS Emulation Using National Health Insurance Claims Data.* Clin Pharmacol Ther, 2025.
46. Voelskow, V., et al., *Trastuzumab in early curative breast cancer: A target trial emulation benchmarked against two randomized clinical trials.* PLoS Med, 2025. **22**(7): p. e1004661.
47. Yiu, Z.Z.N., et al., *Adalimumab Monotherapy vs Adalimumab With Methotrexate for Psoriasis.* JAMA Dermatol, 2025. **161**(7): p. 731-738.
48. Brant, S.B., et al., *Comparative Effectiveness of First-Line Pembrolizumab vs. Chemotherapy in aNSCLC: A Norwegian Population-Based Cohort Study.* Clin Pharmacol Ther, 2025. **117**(4): p. 1123-1130.

49. Himmelreich, J.C.L., et al., *Emulation of ARISTOTLE and ROCKET AF trials in real-world atrial fibrillation patients results in similar efficacy and safety as original landmark trials: insights from the GARFIELD-AF registry.* Open Heart, 2025. **12**(1).
